# The Biodiversity of the Genus *Dictyota*: Phytochemical and Pharmacological Natural Products Prospectives

**DOI:** 10.3390/molecules27030672

**Published:** 2022-01-20

**Authors:** Mohammed I. Rushdi, Iman A. M. Abdel-Rahman, Eman Zekry Attia, Hani Saber, Abdullah A. Saber, Gerhard Bringmann, Usama Ramadan Abdelmohsen

**Affiliations:** 1 Department of Pharmacognosy, Faculty of Pharmacy, South Valley University, Qena 83523, Egypt; mrushdy258@svu.edu.eg (M.I.R.); iman_abdelraheem@yahoo.com (I.A.M.A.-R.); 2 Department of Pharmacognosy, Faculty of Pharmacy, Minia University, Minia 61519, Egypt; eman_zekry@mu.edu.eg; 3 Department of Botany and Microbiology, Faculty of Science, South Valley University, Qena 83523, Egypt; hani.saber@sci.svu.edu.eg; 4 Botany Department, Faculty of Science, Ain Shams University, Abbassia Square, Cairo 11566, Egypt; abdullah_elattar@sci.asu.edu.eg; 5 Institute of Organic Chemistry, University of Würzburg, Am Hubland, 97074 Würzburg, Germany; 6 Department of Pharmacognosy, Faculty of Pharmacy, Deraya University, Universities Zone, New Minia 61111, Egypt

**Keywords:** *Dictyota*, Phaeophyceae, Dictyotaceae, marine macroalgae, brown seaweeds, natural products, bioactivities

## Abstract

Although a broad variety of classes of bioactive compounds have already been isolated from seaweeds of the genus *Dictyota*, most different species are still chemically and biologically unexplored. *Dictyota* species are well-known brown seaweeds belonging to the Dictyotaceae (Phaeophyta). The phytochemical composition within the genus *Dictyota* has recently received considerable interest, and a vast array of components, including diterpenes, sesquiterepenes, sterols, amino acids, as well as saturated and polyunsaturated fatty acids, have been characterized. The contribution of these valued metabolites to the biological potential, which includes anti-proliferative, anti-microbial, antiviral, antioxidant, anti-inflammatory, and anti-hyperpigmentation activities, of the genus *Dictyota* has also been explored. Therefore, this is the most comprehensive review, focusing on the published literature relevant to the chemically and pharmacologically diverse biopharmaceuticals isolated from different species of the genus *Dictyota* during the period from 1976 to now.

## 1. Introduction

**Figure 1:**
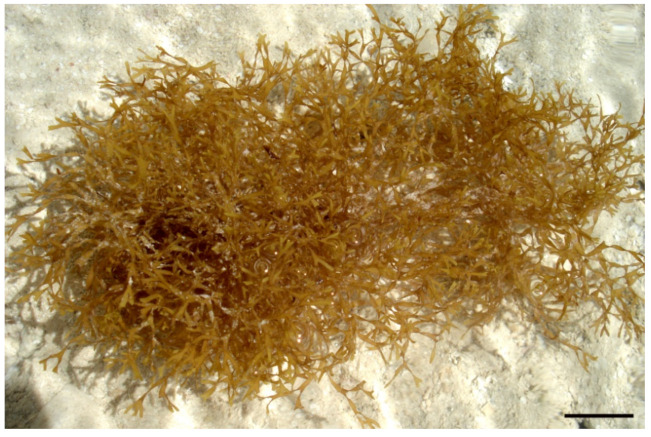
*Dictyotadichotoma* specimens from the Egyptian coastal water of Hurghada City, the Red Sea. Scale bar = 2 cm.

Marine natural products are characterized by the diversity of their chemical structures, displaying a wide spectrum of biological activities. The Red Sea is one of the most important marine hotspots comprising high seaweed biodiversity [1]. Several marine algae, particularly seaweeds, are biogeographically native to the Red Sea, but only a few species have been chemically examined during the past decades. Seaweeds have a growing number of successful applications in the food, medicine, and cosmetic industries, which increases the importance of evaluating their chemical composition [2]. Species of the genus *Dictyota* J.V. Lamouroux, as representatives of these seaweeds, are characterized by the following taxonomic features: flattened thalli, ribbon-like, erect or prostrate, with smooth dentate, crenulate, and ciliate margins; thallus attachment is fulfilled by means of uniseriate, multicellular branching, and hyaline rhizoids that may be divided terminally into fixing disk stoloniferous holdfasts, which may be present or absent; dichotomous orrarely falcate branching; obtuse, rounded, apiculate, or acute apices; Phaeophyceae hairs and superficial proliferations may be present or absent; the thallus differentiates into a cortex and a medulla, and the relative number of layers is variable; the scattered arrangement of sporangia throughout the thallus makes them easily separated from oogonia; the antheridia are prearranged in ellipse white sori. Ecologically, the genus *Dictyota* is primarily growing in tropical and subtropical marine waters on rocky reefs. In 1809, it was discovered by J.V. Lamouroux. So far, 222 different species are documented in the algae database; out of them, 98 species have been accepted taxonomically [3]. *Dictyota* is the richest genus of the family Dictyotaceae and produces a significant number of secondary metabolites, especially diterpenes. Many members of this family produce cyclic diterpenes, unique in the structural variety of marine natural products. The family Dictyotaceae has given rise to a great number of diterpenes generally grouped in three types: xenicanes, extended sesquiterpenes, and dolabellanes. One of the main seaweeds, named *Dictyota dichotoma* (Figure 1), grows on the coral reefs of Hurghada (the Red Sea, Egypt). The species *D. dichotoma* is the most widely distributed member of this family. It has been extensively studied, affording diterpenes of the three mentioned groups, although the studies have noted a wide range of variations among its constituents depending upon time and location of collection [4]. The genus *Dictyota* has recently gathered substantial attention from many researchers thanks to its economic significance during the past decade, represented in its potential as antibiofouling, animal feed, and pharmaceutical agents [5,6]. Some regions, including Hawaiian, Caribbean, and Malayan-Indonesian regions, locally consume *Dictyota*. Its application in the biofuel industry is attributed to its high contents of lipids and fatty acids [7]. *Dictyota* is also well known due to its high contents of sulfated polysaccharides such as fucans and fucoidans, which are responsible for great therapeutic applications such as immunomodulatory, antitumor, vaccine adjuvant, anti-inflammatory, antiprotozoal, nticoagulant, antiviral, antilipemic, and antimicrobial. It also plays a role in drug delivery and tissue engineering application [8]. Diterpenes have been of greater interest than other classes in literature survey; thus, Chen et al. reviewed diterpenes isolated from the genus *Dictyota* until 2018. Bogaert et al. produced an overview on the taxonomy, anatomy, cytology, genetic data, life history, chemical constituents, nutritional value as well as the economic and ecological significance of *Dictyota* species [7]. Accordingly, this review concentrates on the published literature relevant to the chemically and pharmacologically diverse biopharmaceuticals isolated from different species of the genus *Dictyota* during the period from 1976 to now.

## 2. The Pharmacological and Phytochemical Potential of the Genus *Dictyota*

The current review concentrates on studies on the chemical composition of either isolated compounds or extracts and their bioactivities, whenever possible, afforded from different species of genus *Dictyota* [8,9].

### 2.1. Dictyota acutiloba J. Agardh 1848 

**Figure 2:**
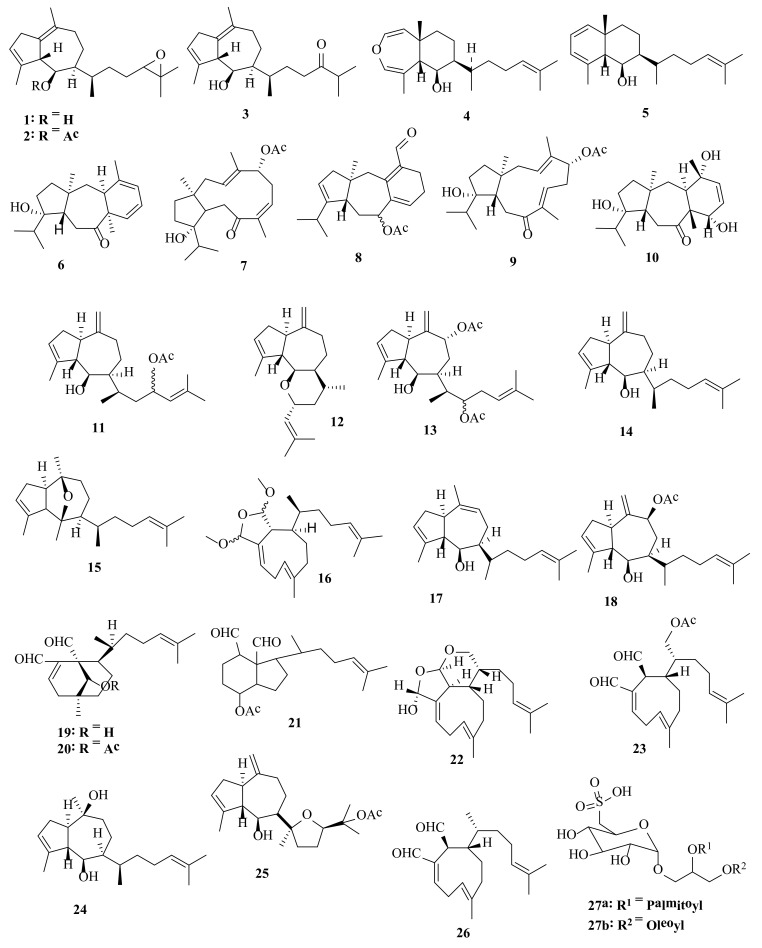
Chemical structures of compounds **1**–**27**. Diterpenes **1**–**5** were isolated from *D. acutiloba*, diterpenes **6**–**10** were isolated from *D. bartayresiana*, diterpenes **11**–**16** were isolated from *D. binghamiae*, diterpenes **17**–**21** were isolated from *D. caribaea*, and diterpenes **22**–**26** and sulfonoglycolipid **27** were isolated from *D. ciliolata*. The configuration of all stereocenters in each compound is shown as given in the literature.

Acutilol A (**1**), acutilol A acetate (**2**), and acutilol B (**3**) were detected in *D. acutiloba*, collected from Kauai (Hawaii) [10]. These acutilols diterpenes, compounds **1**–**3**, are based on the common pachydictyane carbon skeleton but possess unusual Δ^1,10^ double bonds and act as potent agents against both temperate and tropical herbivorous fishes and sea urchins, suggesting that these molecules provide an effective chemical defense. Diterpenes likewise identified in *D. acutiloba*, yet collected from the Kahala and Ala Moana reefs (Hawaii), were the diterpenes dictyoxepin (**4**) and dictyolene (**5**) (Figure 2) [11]. The crude chloroform and crude acetone extracts *of D. acutiloba* from Rameswaram Island (India) were found to have anti-microbial activities against *Bacillus subtilis*, *Salmonella typhi*, Methicillin-resistant *Staphylococcus aureus* (MRSA)*, Staphylococcus aureus*, *Klebsiella pneumoniae*, *Pseudomonas aeruginosa*, *Enterobacter* sp., *Candida albicans*, and *Aspergillus niger* (mean inhibition zone from 9 ± 0.02 to 23 ± 0.031 mm) and no antimicrobial activity was detected in crude methanolic or hexane extracts [12]. 

### 2.2. Dictyota bartayresiana J.V. Lamouroux 1809 

9-Hydroxydolabelladien-6-one (**6**), 5-acetoxy-12-hydroxydolabell-3,7Z-dienone (**7**), 9-acetoxydolabellatrien-16-al (**8**), 5-acetoxy-12-hydroxydolabell-3,7E-dienone (**9**), and trihydroxydolasta-2-en-6-one (**10**) (Figure 2) are diterpenes detected in *D. bartayresiana*, collected in the Gulf of Mannar of the Indian Ocean [13]. The antifungal effects of silver nanoparticles (50 μL) were investigated, using an aqueous extract of brown seaweed *D. bartayresiana* collected from the Mandapam coastal region, Gulf of Mannar (India), against *Mucor circinelloids* and *Fusarium dimerum* (mean zone of inhibition: 19 and 12 mm, respectively) [14]. Steroids, alkaloids, phenolic compounds, cardiac glycosides, flavonoids, saponins, tannins, and amino acids were identified in the phytochemical analysis of various extracts of *D. bartayresiana*, collected from Rasthacaud coastal waters, Kanyakumari District, Tamil Nadu, India. It was observed that the methanolic extract of *D. bartayresiana* expressed a maximum zone of inhibition against *Escherichia* *coli* (9.4 ± 0.2 mm). A crude methanolic extract of *D. bartayresiana* showed the highest larval mortality (median lethal dose, LC_50_ = 166.33 mg/L and 90% mortality LC_90_ = 265.69 mg/L) against *Culex quinquefasciatus*. Similarly, the methanolic extract of *D. bartayresiana* displayed the highest cytotoxicity, with LC_50_ and LC_90_ values of 202.63 and 354.24 mg/L, respectively, against *Artemia salina* [15]. The antioxidant potential of the methanolic extract of *D. bartayresiana* J.V. Lamouroux (Phaeophyta), collected from the Mandapam coastal region of the Gulf of Mannar (southeast coast of India), was determined, for which the radical-scavenging 2,2-diphenyl-1-picrylhydrazyl assay (DPPH), the ferric reducing antioxidant power assay (FRAP), and the superoxide-scavenging antioxidant capacity assay were performed. The methanolic extract of *D. bartayresiana* showed a DPPH radical-scavenging activity (the half maximal inhibitory concentration being IC_50_ = 8.2 µg/mL), an ascorbic acid equivalent (IC_50_ = 3 µg/mL) and FRAP assay values of the extract, with ascorbic acid (IC_50_ = 9.4 and 5.6 µg/mL) as the reference standard. An eight-fold increase in scavenging superoxide radicals was observed; the IC_50_ value (513.84 µg/mL) was comparable with that of the reference standard quercetin (IC_50_ = 67.8 µg/mL) [16]. The bio-synthesized SiO_2_–ZnO nanocomposites of *D. bartayresiana*, harvested from the Mandapam region (India), revealed promising potential against the HT29 cancer cell line, inhibited bacterial colonies of urinary tract infection pathogens, and showed promising antioxidant activity [17].

### 2.3. Dictyota binghamiae J. Agardh 1894

Dictyol G acetate (**11**), dictyoxide A (**12**), dictyotriol A diacetate (**13**), pachydictyol A (**14**), dictyoxide (**15**), and dimethoxy dictyodial (**16**) (Figure 2) were detected in *D. binghamiae*, collected from Barkley Sound (British Columbia) [18].

### 2.4. Dictyota caribaea Hörnig and Schnetter 1992

The cytotoxic and anti-leishmanial activities of crude extracts of *D. caribaea* collected in the Gulf of Mexico and the Caribbean coast have been reported. The study revealed the presence of fucoidan, a humofucan with fucose as the neutral sugar. The sulfated polysaccharide-treated mice with 25 and 50 mg/kg/day depicted Sarcoma 180 tumor growth inhibition of 40% and 51%, respectively, compared to negative control group. Sulfated polysaccharides treatment induced spleen weight increasing compared to the negative control group of 40% and 70% on mice treated with 25 and 50 mg/kg/day, respectively, along with intense white pulp disorganization. These results combined support an immunostimulant effect related to the in vivo antitumor effect of *D. caribaea* sulfated polysaccharides [19].

Sulfated polysaccharides isolated from *D.* *caribaea*, collected from Rio de Janeiro (Brazil), were tested for their antitumor effects. Antiproliferative activity of *D. caribaea* was tested against colon cancer (HCT 116) and metastatic melanoma (B16–F10) cell lines. The antitumor effect was evaluated on Swiss mice transplanted with sarcoma 180 tumor and treated by intraperitoneal injection during 7 days with saline or *D. caribaea* (25 and 50 mg/kg/animal). *D. caribaea* did not exhibit cytotoxicity in vitro, but mice treated with *D. caribaea* showed up to 50% tumor growth inhibition. *D. caribaea* treatment induced an increase in spleen weight, along with an intense white-pulp disorganization. Furthermore, *D. caribaea* did not exert hepatic toxicity, nephrotoxicity, or leukopenia, but it did induce an increase in platelets count [20]. The diterpenes pachydictyol A (**14**), isopachydictyol A (**17**), dictyol B acetate (**18**), dichotomanol (**19**), dichotomanol acetate (**20**), and cycloxenianol acetate (**21**) (Figure 2) were discovered in *D. caribaea*, collected from the Caribbean coast [21,22].

### 2.5. Dictyota ciliolata Sonder ex Kützing 1859

Ciliolatale (**22**), 17-acetoxydictyodial (**23**), dictyol C (**24**), dictyol H (**25**), and dictyodial (**26**) (Figure 2) were detected in *D. ciliolata*, collected from Oualidia Lagoon (Morocco). Of these, 17-acetoxydictyodial (**23**) displayed mild antifungal activity against *C. albicans* (MIC = 50 mg/mL) [23]. The sulfonoglycolipid 3-*O*-(6′-deoxy-6′-sulfo-α-D-glucopyranosyl)-1-*O*-oleoyl-2-*O*-palmitoylglycerol (**27**) was found in *D. ciliolata* [24]. Pachydictyol A (**14**) and dictyol B acetate (**18**) were isolated from *D. ciliolata*, collected from the Caribbean coast of the Yucatan peninsula. Pachydictyol A (**14**) reduced activity against all cancer cell lines tested—the ubiquitous keratin-forming tumor (KB), human epidermoid carcinoma, (Hep-2), human breast cancer (MCF-7), and human cervical cancer (SiHa)—whereas dictyol B acetate (**18**) showed cytotoxic activity only against Hep-2 (the 50% cytotoxic concentration, CC_50,_ varying between 14.8 and 41.2 mg/mL) [25].

### 2.6. Dictyota coriacea (Holmes) I.K. Wang, H.S. Kim and W.J. Lee 2004 

**Figure 3:**
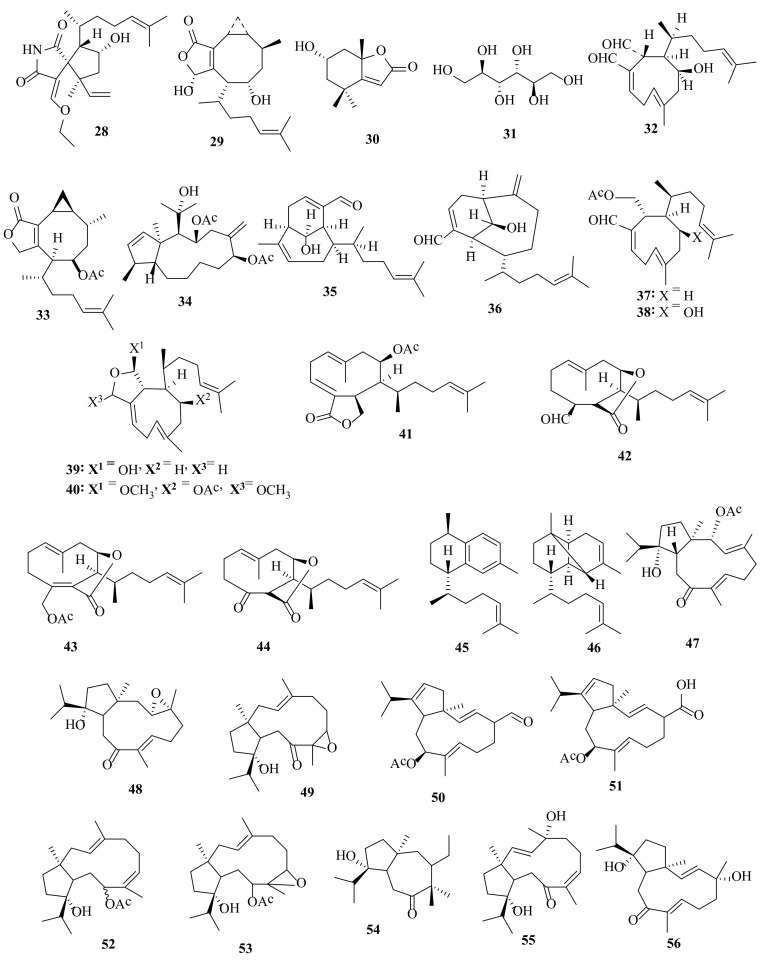
Chemical structures of compounds **28**–**56**. The spirosuccinimide alkaloid **28**, thediterpenes **29**–**30**, and the sugar **31** were isolated from *D. coriacea*, the diterpenes **32**–**36** were isolated from *D. crenulata*, and the diterpenes **37**–**56** were isolated from *D. dichotoma.* The configuration of all stereocenters in each compound is shown as given in the literature.

Dictyospiromide (**28**) is a spirosuccinimide alkaloid, discovered in *D. coriacea* from the coast of Nanji Island, Wenzhou, Zhejiang Province (China). It has antioxidant properties, associated with activation of the Nrf2/ARE signaling pathway [26]. 1,9-Dihydroxycrenulide (**29**), epiloliolide (**30**), and D-mannitol (**31**) (Figure 3) were isolated from the ethanolic extract of *D. coriacea*, collected from the coasts of Jeju Island (Korea). The melanin synthesis inhibition activities were evaluated using B16F10 melanoma cells for the isolates. Compared with the positive control, arbutin, 1,9-dihydroxycrenulide (**29**) and epiloliolide (**30**) (Figure 3) exhibited more potency for treating hyperpigmentation and effective components of whitening cosmetics, showing 27.8 and 22.6% inhibition activities, respectively, at a substrate concentration of 30 μg/mL [27]. 1,9-dihydroxycrenulide (**29**) and epiloliolide (**30**) isolated from *D. coriacea* extract caused an increase in the proliferation of dermal papilla cells (DPCs). When isolated rat vibrissa follicles were treated with 1,9-dihydroxycrenulide (**29**) and epiloliolide (**30**) for 21 days, the hair-fiber lengths for the vibrissa follicles increased. Several solvent fractions of *D. coriacea* were examined for the activity of 5α-reductase, which converts testosterone to dihydrotestosterone (DHT), a main cause of androgenetic alopecia. The results revealed the significant decrease in 5α-reductase activity they caused, while 1,9-dihydroxycrenulide (**29**) and epiloliolide (**30**) scarcely inhibited 5α-reductase activity. In addition, the *D. coriacea* extract and several solvent fractions of *D. coriacea* extract could not act as a KATP channel opener, which could be a contributory factor in the effect on hair growth. *D. coriacea* extract and 1,9-dihydroxycrenulide (**29**) and epiloliolide (**30**), principals of *D. coriacea*, have the potential to treat alopecia via the proliferation of DPCs [28].

The ethanolic extract, as well as the *n*-hexane and ethyl acetate fractions of *D. coriacea*, collected from the sea adjacent to Jeju Island, displayed a dependently inhibitory effect on tyrosinase activity and melanin content in B16F10 cells. The ethanolic extract and its fractions showed an inhibitory effect on tyrosinase and human tyrosinase-related protein-1 (TRP-1) gene transcription but did not exert an inhibitory effect on human tyrosinase-related protein-2 (TRP-2) gene transcription. Moreover, the *n*-hexane and ethyl acetate fractions dose-dependently inhibited the production of NO in RAW 264.7 cells. These results suggest that the extract of *D. coriacea* could be used as a functional biomaterial in developing a skin-whitening agent that has anti-inflammatory activity [29].

### 2.7. Dictyota crenulata J. Agardh 1847

4β-Hydroxydictyodial A (**32**) was detected in *D. crenulata*, collected at Kualoa Beach Park, Oahu, Hawaii [30]. A bicyclic cyclopropane-containing diterpenoid, named acetoxycrenulide (**33**), was found in *D. crenulata*, collected from the Gulf of California (Mexico). Dictyocrenulol (**34**) was identified in *D. crenulata*, collected from Hanga Roa (Chili) [31]. β-Crenulal (**35**) and sanadaol (**36**) (Figure 3) were discovered in *D. crenulata*, harvested from Japan [32]. Pachydictyol A (**14**), isopachydictyol A (**17**), dictyol C (**24**), dictyodial (**26**), 4β-hydroxydictyodial A (**32**), prenylated guaiane, and xenia diterpenes were isolated from the crude extract of the Brazilian *D. crenulata* [33]. Both (−)- and (+)-sanadaol (**36**) were synthesized stereoselectively from a chiral bicyclo[2.2.2]octane derivative prepared from D-mannitol [34].

### 2.8. Dictyota dichotoma (Hudson) J.V. Lamouroux 1809

*D. dichotoma* was the most valuable seaweed due to its relatively high protein content of 7.28 ± 0.25%, moderate carbohydrate content of 25.35 ± 0.32%, and highest pigment and antioxidant contents [35]. In *D. dichotoma*, collected from Peter the Great Bay, Sea of Japan (Russia), the dictyota D-glucan laminaran (DdL) and the dictyota D-glucan fucoidan (DdF) were detected. DdF is a sulfated (28.9%) and acetylated heteropolysaccharide containing fucose, galactose, mannose, and glucose (57.9, 20.4, 12.4, and 9.2 mol%, respectively). Laminaran is a 1,3;1,6-β-D-glucan with the main chain built up from 1,3-linked D-glucose residues [36].

There can be 6-*O*-branches and β-(1→6) intrachain links in the main chain. Sulfated (43.7%) laminaran DdLs were obtained from DdL by chemical sulfation.

The sulfates were found to occur at C2, C4, and C6 of the glucose residues. The anticancer effects of DdF, DdL, and DdLs (200 μg/mL) were measured in vitro on colon cancer cells (HCT-116, HT-29, and DLD-1). The effect of polysaccharides (40 μg/mL) on colony formation of DLD-1 cancer cells after irradiation was investigated. All polysaccharides showed a synergistic effect with X-ray irradiation against cancer cells, decreasing the amount and size of cancer cell colonies [36].

The laminarans are neutral, water-soluble β-D-glucans possessing potent immunomodulating, radioprotective, and anticancer activities. The in vitro anticancer, radioprotective, and radiosensitizing activities of laminaran from *D. dichotoma* and its sulfated derivative were investigated. The native and sulfated laminarans by themselves at non-toxic doses possessed significant anticancer activity against melanoma cells. Both polysaccharides protected normal epidermal cells, while only sulfated laminaran was able to sensitize melanoma cells to X-ray irradiation, resulting in significant inhibition of cell proliferation, colony formation, and migration of cancer cells [37].

The molecular mechanism of this action was found to be related to the inhibition of matrix metalloproteinase-2 (MMP-2) and matrix metalloproteinase-9 (MMP-9) proteinases activity and to the down-regulation of kinases phosphorylation of the ERK1/2 signaling cascade [37].

The galactofucan-rich fractions, obtained from *D. dichotoma* thalli exhibited moderate inhibitory effects against *Herpes simplex* virus (HSV-1) and Coxsackie virus (CVB3) [38]. The ethanolic extract of *D. dichotoma* possessed active compounds for the development of larvicidal activity against 4th instars larvae of the yellow-fever mosquito, *Aedes aegypti*. The extract of *D. dichotoma* revealed that the minimum level of an LC_50_ value was 0.0683 ± 0.0084 μg/mL.

The preliminary phytochemical constituents showed the presence of saponins, steroids, terpenoid, phenols, protein, and sugars [39]. Hexadecanoic acid, tetradecanoic acid, 2-hydroxyhexadecanoic acid, palmitic acid, elaidic acid, stearic acid, *cis*-11,14,17-eicosatrienoic acid, and erucic acid were isolated from *D. dichotoma*, collected at Pulau Nunuyan Laut, Sandakan, Sabah, with the contents of the co-occurring poly- and mono-unsaturated fatty acids being higher than the saturated fatty ones [40].

Diacylglycerol hydroxymethyl trimethyl-β-alanine (DGTA), a recently identified betaine lipid [41], was found as a major lipid component in *D. dichotoma*, collected at the seashore near Tokyo, Japan. For the fatty acids of DGTA in *D. dichotoma*, the major components were palmitic acid 16:0, linoleic acid 18:2, and arachidonic acid 20:4ω6 [41].

For *D. dichotoma*, collected from the Western seacoast of Yemen, anticancer and antioxidant activities were detected. The chloroform fraction of the *D. dichotoma* displayed the highest cytotoxic activity and was most effective against MCF-7, human prostate cancer cells (PC-3), and colorectal adenocarcinoma cells (CACO), (IC_50_ = 1.93 ± 0.25, 2.2 ± 0.18, and 2.71 ± 0.53 μg/mL, respectively). The petroleum ether fraction was also effective, particularly against MCF-7 and PC-3 (IC_50_ = 4.77 ± 0.51 and 3.93 ± 0.51 μg/mL, respectively), whereas the activity of the ethyl acetate fraction was more pronounced against HepG2 and CACO (IC_50_ = 5.06 ± 0.21 and 5.06 ± 0.23 μg/mL, respectively). Of all the extracts tested, the crude methanolic extract of the algae exhibited the best antioxidant potential (IC_50_ = 204.6 ± 8.3 μg/mL). Doses as high as 5000 mg/kg body weight of *D. dichotoma* methanolic extracts were safe and well tolerated by rats [42].

In *D. dichotoma*, collected from Oshoro Bay, Hokkaido, acetyldictyolal (**37**), hydroxyacetyldictyolal (**38**), isodictyohemiacetal (**39**), and dictydiacetal (**40**), i.e., cyclononane diterpenes were detected [43].

4-Acetoxydictyolactone (**41**), dictyotalide A (**42**), dictyotalide B (**43**), and nordictyotalide (**44**) were discovered in *D. dichotoma*, collected from the coast of Nakijin, Okinawa, with significant cytotoxic activity against B16 melanoma cells [44].

The diterpenes *ent*-erogorgiaene (**45**) and 1,5-cyclo-tetrahydroerogorgiaene (**46**) were identified in the Russian far-eastern population of *D. dichotoma* [45].

Acetoxy-hydroxy-dolabella-3,7-dien-9-one (**47**), 3,4-epoxy-hydroxy-dolabella-7-en-9-one (**48**), 7,8-epoxy-hydroxy-dolabella-3-en-9-one (**49**), 9-acetoxydolabella-3,7,12-trien-16-al (**50**), 9-acetoxydolabellatrien-16-oic acid (**51**), 9-acetoxydolabella-3,7-dien-12-ol (**52**), 9-acetoxy-7,8-epoxydolabella-3-en-12-ol (**53**), 9-hydroxydolest-1,3-dien-6-one (**54**), 4,12-dihydroxydolabella-2,7Z-dien-9-one (**55**), 4,12-dihydroxydolabella-2,7E-dien-9-one (**56**) (Figure 3), 12-hydroxydolabella-3,7E-dien-9-one (**57**), 12-hydroxydolabella-3,7Z-dien-9-one (**58**), 4,12-dihydroxydolabellan-2,6-dien-9-one (**59**), 4,12-dihydroxydolabellan-2,6-dien-9-one (**60**), 12Z-hydroxydolabella-3,6-dien-9-one (**61**), and 12E-hydroxydolabella-3,6-dien-9-one (**62**) were found in *D. dichotoma* collected from the Indian Ocean [46].

**Figure 4:**
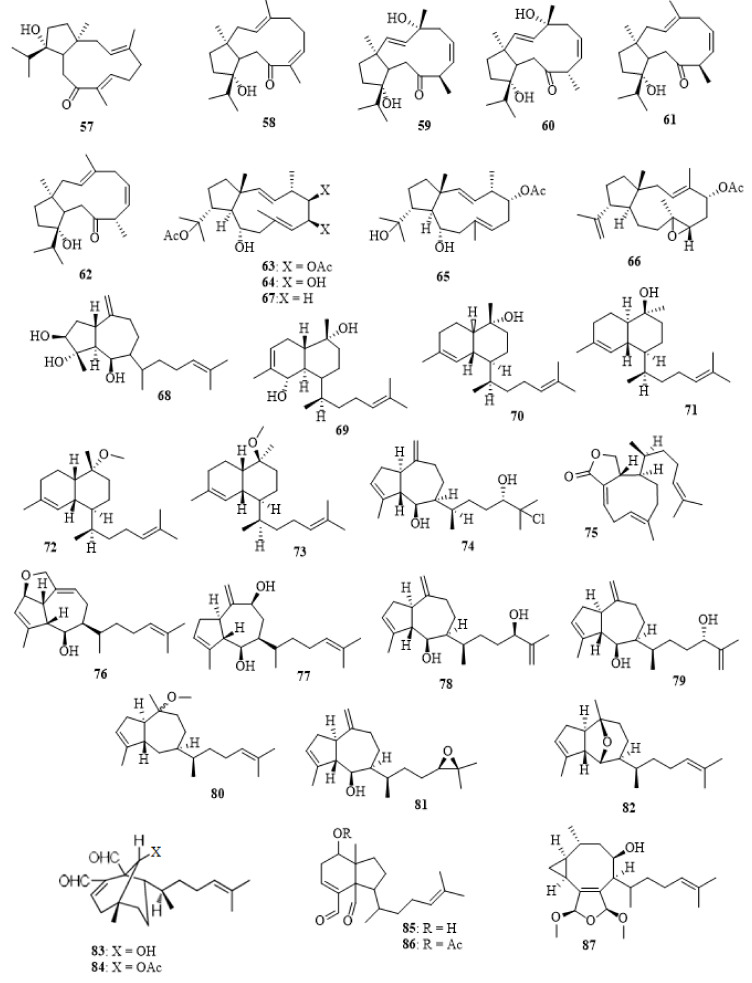
Chemical structures of compounds **57**–**87**. The diterpenes **57**–**87** were isolated from *D. dichotoma*. The configuration of all stereocenters in each compound is shown as given in the literature.

Isopachydictyol A (**17**), 5,6,18-triacetoxy-hydroxy-dolabelladiene (**63**), 18-acetoxy-10-hydroxy-2,7-dolabelladiene (**64**), 5-acetoxy-dihydroxy-2,7-dolabelladiene (**65**), 7,8-epoxy-3,18-dolabelladiene (**66**), 18-acetoxy-2,7-dolabelladiene (**67**), and dictyotatriol A (**68**) (Figure 4) were found in *D. dichotoma* collected from the intertidal zone of Cortadura (Cadiz, Spain) [4].

Dictyotin A (**69**), dictyotin B (**70**), dictyotin C (**71**), methoxydictyldiene (**72**) and dictyotin D methyl ether (**73**) (Figure 4), were isolated from *D. dichotoma* collected in Yagachi, Okinawa [47]. The chlorine-containing perhydroazulene diterpene dictyol J (**74**), dictyolactone (**75**), and sanadaol (**36**) were identified in the ethanolic extract of *D. dichotoma* harvested in Japan. Dictyolactone (**75**) showed the highest algicidal activity against red-tide phytoplanktons. The isolated compounds **36** and **74**–**75** were assayed for their algicidal activity against the three representative harmful algal bloom (HAB) species, *Heterosigma* *akashiwo*, *Karenia mikimotoi*, and *Alexandrium catenella*. All these compounds showed high (>95%) algicidal activity against *H. akashiwo* and *K. mikimotoi* at a dose of 10–20 mg/mL, with dictyolactone (**75**) being the most active one. Interestingly, dictyolactone (**75**) also displayed moderate activity (41.5 ± 8.2% at 10 mg/mL) against the dinoflagellate *Alexandrium catenella*, while the other compounds and the known algicidal agent α-linolenic acid were totally inactive to this species [48]. Dictyol A (**76**) and dictyol B (**77**), two diterpene alcohols with a hydroazulene ring system, were detected in *D. dichotoma* collected from the Italian coast [49].

**Figure 5:**
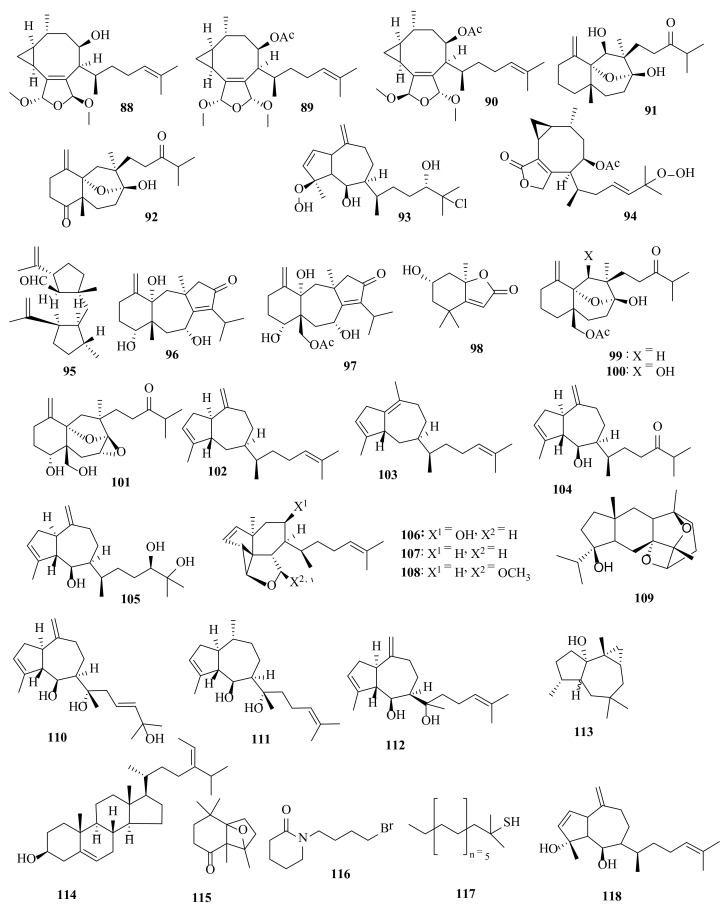
Chemical structures of compounds **88**–**118**. The diterpenes **88**–**113** and **118**, sterol **114**, oxatricyclic derivative **115**, halogenated derivative **116**, and thiol derivative **117** were isolated from *D. dichotoma*. The configuration of all stereocenters in each compound is shown as given in the literature.

Dictyol F (**78**), epidictyol F (**79**), dihydromethoxy-pachydictyol A (**80**), 14,15-epoxy-pachydictyol A (**81**), and 2,6-cycloether pachydictyol A (**82**) were identified in the methanolic extract of *D. dichotoma*, harvested at Oshoro Bay, Hokkaido [50]. 9*R*-Hydroxydichotoma-2,14-dien-19,20-diol (**83**), 9*R*-acetoxydichotoma-2,14-dien-19,20-diol (**84**), 7-hydroxy-2,6-cycloxenicadien-18,19-diol (**85**), and 7-acetoxy-2,6-cycloxenicadien-18,19-diol (**86**) were discovered in the dichloromethane extract of the Australian *D. dichotoma* [51]. Likewise found in *D. dichotoma* were crenulacetal A (**87**) (Figure 4), crenulacetal B (**88**), crenulacetal C (**89**), and crenulacetal D (**90**) [52]. The seco-dolastanes dichotone (**91**) and dichotodione (**92**) were isolated from *D. dichotoma* from Karachi (Pakistan) [53]. Dictyohydroperoxide (**93**), hydroperoxyacetoxycrenulide (**94**) (Figure 5), and the diterpenoid hydroperoxide acetoxycrenulide (**33**) were found in the Russian *D. dichotoma* [54].

Seco-fusicoccin-type diterpene dictymal (**95**) was detected in *D. dichotoma*, collected from Oshoro Bay, Hokkaido [55]. In *D. dichotoma* collected from the Karachi coast, the dolastane-diterpenoids, dichotenone A (**96**), dichotenone B (**97**), and loliolide (**98**) were identified (Figure 5) [56]. Dichotenol A (**99**), dichotenol B (**100**), and dichotenol C (**101**), all C-16 oxidized seco-dolastanes, were discovered in *D. dichotoma*, collected from the Karachi coast of the Arabian Sea [57]. Hydroazulenoid diterpenes, dictytriene A (**102**), dictytriene B (**103**), dictyone (**104**), and dictytriol (**105**) (Figure 5) were isolated from a Japanese *D.* *dichotoma* [58]. The tricarbocyclic cycropropanoid diterpenes tricyclodictyofuran A (**106**), tricyclodictyofuran B (**107**), and tricyclodictyofuran C (**108**) were detected in the methanolic extract of *D. dichotoma*, collected from Oshoro Bay, Hokkaido (Figure 5) [59].

Dictyoxetane (**109**) was isolated from *D. dichotoma*, collected from the Indian Ocean [60]. Pachydictyol B (**110**) and pachydictyol C (**111**) were isolated from the dichloromethane extract of *D. dichotoma* collected from the Red Sea coast of Egypt, along with the known metabolites, pachydictyol A (**14**), dictyol E (**112**), *cis*-africanan-1α-ol (**113**), fucosterol (**114**), tetrahydrothiophen-1,1-dioxide, and poly-β-hydroxybutyric acid. β-Bourbonene and nonanal, along with three hydrocarbons as well as five fatty acids and their simple derivatives were detected in the GC–MS analysis of the nonpolar fractions. GC–MS analysis of the unsaponifiable algal petroleum ether extract revealed the presence of eight further compounds, including 2,2,6,7-tetramethyl-10-oxatricyclo[4.3.0.1(1,7)]decan-5-one (**115**), *N*-(4-bromo-*n*-butyl)-piperidin-2-one (**116**) and *tert*-hexadecanethiol (**117**) (Figure 5). The crude algal extract was potently active against the breast carcinoma tumor cell line, MCF7 (IC_50_ = 0.6 µg/mL); pachydictyol B (**110**) and dictyol E (**112**) showed weak antimicrobial properties, while the other compounds were inactive. Pachydictyol B (**110**) and pachydictyol C (**111**) demonstrated a weak and unselective cytotoxicity against twelve human tumor cell lines with a mean IC_50_ of >30.0 µM [61]. Dictyol B acetate (**18**) and dictyotadiol (**118**) (Figure 5) are diterpenes from *D. dichotoma* [62].

**Figure 6:**
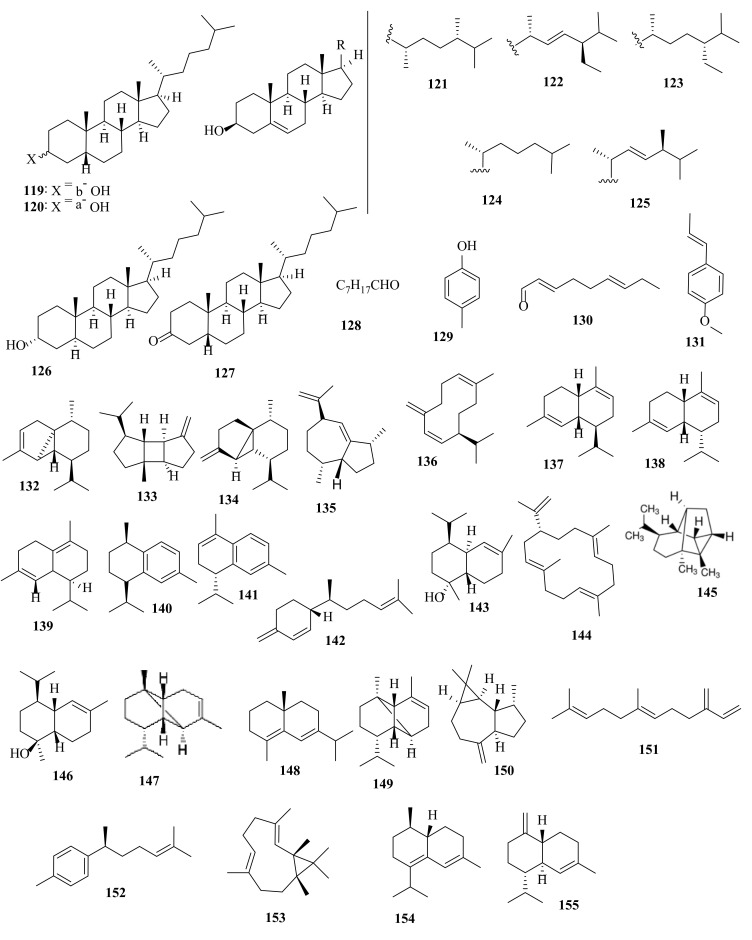
Chemical structures of compounds **119**–**155**. Sterols **119**–**127**, aldehydes **128** and **130**, aromatic derivatives **129**, **131,** and **152**, diterpenes **132**–**133**, sesquiterpenes **134**–**150** and **153**–**154**, and hydrocarbon **151** were isolated from *D. dichotoma*. The configuration of all stereocenters in each compound is shown as given in the literature.

Sterols such as methyl-22-dehydrocholesterol, fucosterol (**114**), coprostanol (**119**), epicoprostanol (**120**), campesterol (**121**), stigmasterol (**122**), β-sitosterol (**123**), cholesterol (**124**), brassicasterol (**125**), cholestanol (**126**), and 5β-cholestan-3-one (**127**) (Figure 6) were discovered in the Malaysian *D. dichotoma* and tested for antifouling activity against *Vibrio alginolyticus, V. mimicus, V. parahaemolyticus, P. aeruginosa*, and *B. subtilis.* Coprostanol (**119**), epicoprostanol (**120**), campesterol (**121**), stigmasterol (**122**), and 5β-cholestan-3-one (**127**) (Figure 6) showed strong inhibition towards the selected bacterial strains, with IC_50_ values ranging from 266.3 to 425.8 μg/mL, while fucosterol (**114**) gave no activity for all bacterial strains tested. Epicoprostanol (**120**) was the most active compound; it inhibited two types of bacteria *V. alginolyticus* (269.00 ± 0.06) and *V. mimicus* (314.81 ± 0.07 μg/mL) [63]. Fucosterol (**114**) had many biological activities, such as antiproliferative and antimicrobial [64]. 1-Octanol (**128**), p-cresol (**129**), 2,6-nonadienal (**130**), *trans*-anethole (**131**), α-cubebene (**132**), β-bourbonene (**133**), β-cubebene (**134**), γ-gurjurene (**135**), germacrene D (**136**), α-muurolene (**137**), α-amorphene (**138**), δ-cadenene (**139**), *cis*-calamenene (**140**), α-calacorene (**141**), β-sesquiphellandrene (**142**), α-cadinol (**143**), cembrene (**144**) (Figure 6), and palmitic acid were identified in the essential oil of *D. dichotoma* collected from the west of Algeria to the wilaya of Tipaza. With 10.7%, *trans*-anethole (**131**) was the main component in the respective oils. For extracts of *D. dichotoma*, the dichloromethane extract was the most active one against DPPH radicals, with an IC_50_ value of 0.517 mg/mL, followed by the chloroform extract (IC_50_ = 0.612 mg/mL). The hexane extract of *D. dichotoma* displayed the lowest antiradical activity, with an IC_50_ value of 1.72 mg/mL [65].

Headspace-volatile organic compounds of fresh *D. dichotoma*, collected from the Adriatic Sea, Mala Smokvica Island, were: ethanol, oct-1-en-3-ol, octan-1-ol, decan-1-ol, (2Z)-pent-2-en-1-ol, dimethyl sulfide, benzene, toluene, pent-1-en-3-one, oct-1-en-3-one, 6-methylhept-5-en-2-one, (2E)-pent-2-enal, 3-methylbut-2-enal, (2E)-oct-2-enal, benzaldehyde, pentanal, hexanal, heptanal, nonanal, (2Z)-hept-2-enal, (2E,4Z)-hepta-2,4-dienal, (3E)-octa-1,3-diene, fucoserraten, (2E,4E,6E)-octa-2,4,6-triene, heptadecane, nonadecane, α-cubebene (**132**), β-bourbonene (**133**), β-cubebene (**134**), germacrene D (**136**), α-muurolene (**137**), α-amorphene (**138**), δ-cadenene (**139**), α-calacorene (**141**), α-cadinol (**143**), cycloisosativene (**145**), τ-muurolol (**146**), α-ylangene (**147**), δ-selinene (**148**), α-copaene (**149**), aromadendrene (**150**), (E)-β-farnesene (**151**), α-curcumene (**152**), bicyclogermacrene (**153**), epizonarene (**154**), γ-cadinene (**155**) (Figure 6), γ-muurolene (**156**), germacrene B (**157**), *trans*-cadina-1,4-diene (**158**), and 1,8-cineole (**159**) [66].

**Figure 7:**
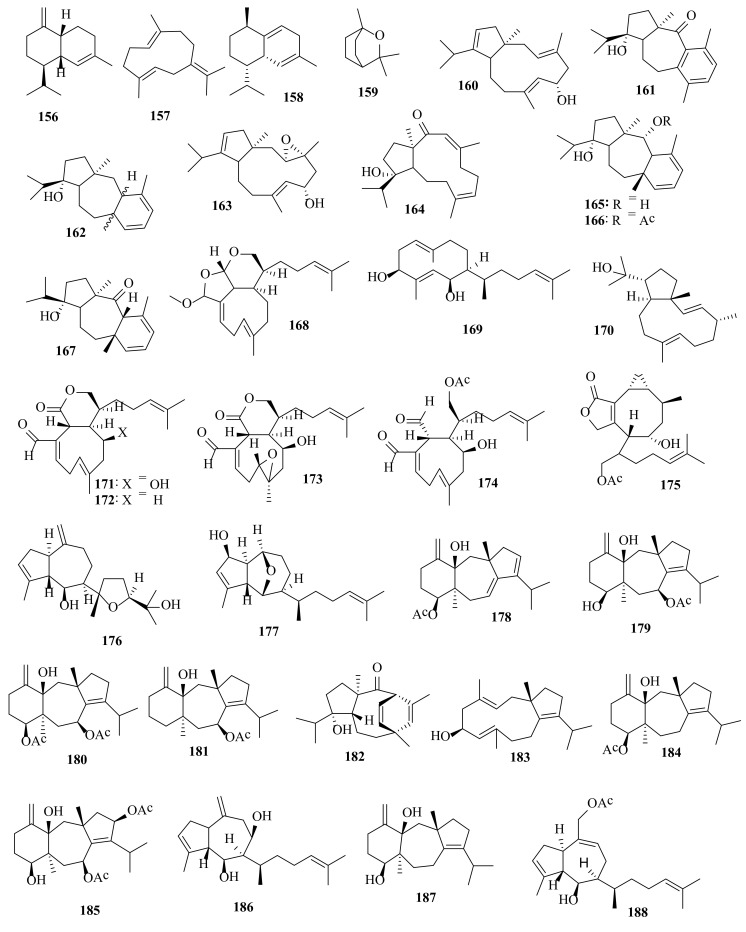
Chemical structures of compounds **156**–**188**. Sesquiterpenes **156**–**159** were isolated from *D. dichotoma*, diterpenes **160**–**182** were isolated from *D. dichotoma* var. *divaricata*, and diterpenes **183**–**188** were isolated from *D. dichotoma* var. *implexa.* The configuration of all stereocenters in each compound is shown as given in the literature.

6-Hydroxydolabella-3,7,12-triene (**160**), 9-hydroxyisodolasta-1,3,5(14)-trienone (**161**), 9-hydroxydolasta-1,3-diene (**162**), 3,4-epoxy-6-hydroxydolabella-7,12-diene (**163**), 12-hydroxydolabella-3Z,7E-dien-2-one (**164**), 9,13-dihydroxydolasta-1,3-diene (**165**), 13-acetoxy-9-hydroxydolasta-1,3-diene (**166**) and 9-hydroxydolasta-1,3-dien-13-one (**167**) (Figure 7) are diterpenes detected in the crude chloroform-methanol extract of *D. dichotoma* var. *divaricata*, collected from the coast of Neil Island in the Andamans [67].

17,18:18,19-Bisepoxyxenic-methoxy-triene (**168**), 3β-hydroxydilophol (**169**), and 18-hydroxy-2,7-dolabelladiene (**170**) (Figure 7) were identified in *D. dichotoma* var. *divaricata* collected from the Great Barrier Reef region of Northern Australia [68]. 4,17-Hydrosyxenic-trienaloic acid lactone (**171**), 17-xenic-trien-1-al-18-oic acid lactone (**172**), epoxyxenic-hydroxydienaloic acid lactone (**173**), 17-acetoxyxenic-4-hydroxy-trien-dial (**174**), 17-acetoxy-4α-hydrocrenulide (**175**), deacetoxydictyol H (**176**), and 2-hydroxydictyoxide (**177**) (Figure 7) were detected in *D. dichotoma* var. *divaricata*, collected from Australia [69].

(3αS,4αR,8S,8αS)-4α-Hydroxy-3α,8α-dimethyl-5-methylidene-1-(propan-2-yl)-3,3α,4,4α,5,6,7,8,8α,9-decahydrobenzo[f]azulen-8-yl acetate (**178**), (3α*S*,4α*R*,8*S*,8α*S*,10*S*)-4α,8-dihydroxy-3α,8α-dimethyl-5-methylidene-1-(propan-2-yl)-2,3,3α,4,4α,5,6,7,8,8α,9,10-dodecahydrobenzo[f]azulen-10-yl acetate (**179**), (3α*S*,4α*R*,8*S*,8α*S*,10*S*)-4a-hydroxy-3α,8α-dimethyl-5-methylidene-1-(propan-2-yl)-2,3,3α,4,4α,5,6,7,8,8α,9,10-dodecahydrobenzo[f]azulene-8,10-diyl diacetate (**180**) and (3α*S*,4α*R*,8α*R*,10*S*)-4α-hydroxy-3α,8α-dimethyl-5-methylidene-1-(propan-2-yl)-2,3,3α,4,4α,5,6,7,8,8α,9,10-dodecahydrobenzo[f]azulen-10-yl acetate (**181**) (Figure 7) are tricyclic diterpenoids of the dolastane ring system detected in *D. dichotoma* var. *divaricata*, collected from the Caribbean Sea [70].

Divarinone (**182**), a tricyclic diterpene, was found in *D. dichotoma* var. *divaricata* collected from the Indian Ocean [71]. Pachydictyol A (**14**), isopachydictyol A (**17**), dictyol C (**24**), dolabellatrienol (**183**), amijiol acetate (**184**), dolastane amijiol-7,10-diacetate (**185**), 8β-hydroxy-pachydictyol A (**186**), and amijiol (**187**) (Figure 7) were identified in *D. dichotoma* var. *implexa*. collected from the Red Sea. Among these compounds, amijiol acetate (**189**) and dolastane amijiol-7-10-diacetate (**161**) had potent activity against DNA damage, cytotoxicity against WI-38, HepG2, and MCF-7 cell lines, and showed antioxidant activity using ABTS and erythrocyte hemolysis [72].

**Figure 8:**
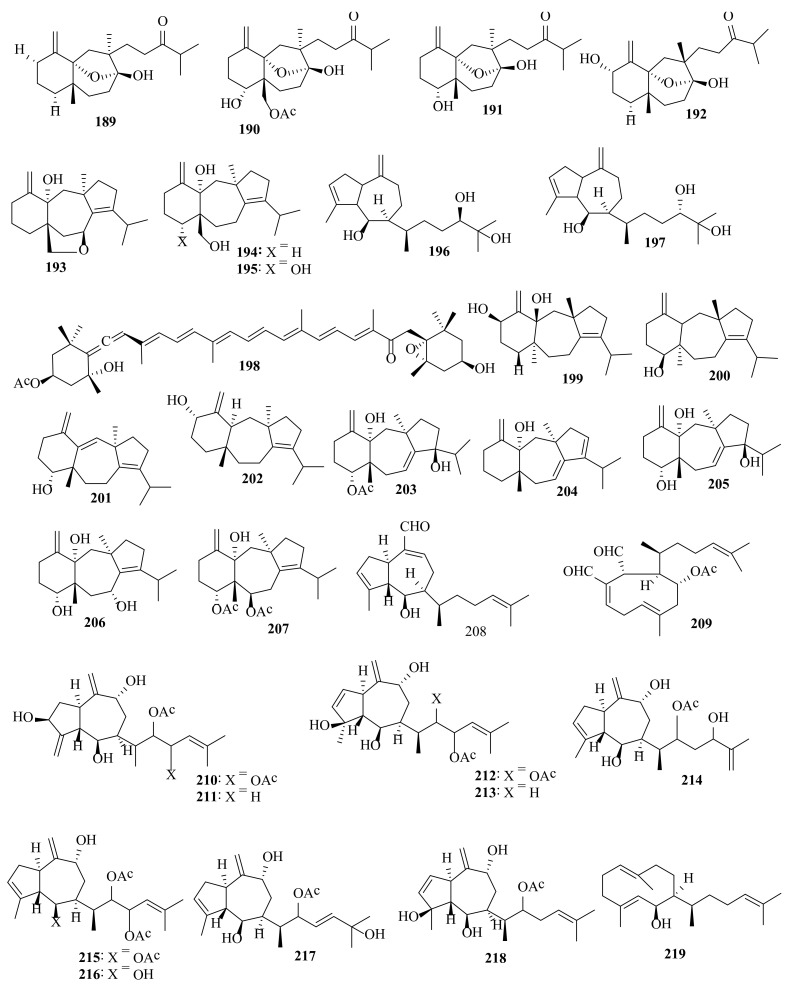
Chemical structures of compounds **189**–**219**. Diterpenes **189**–**197** and carotenoid **198** were isolated from *D. dichotoma* var. *indica*, diterpenes **199**–**209** were isolated from *D. dichotoma* var. *linearis*, and diterpenes **210**–**219** were isolated from *D. dichotoma* var. *volubilis*. The configuration of all stereocenters in each compound is shown as given in the literature.

Pachydictyol A (**14**), dictyol B acetate (**18**), and dictyol I acetate (**188**) (Figure 7) were found in *D. dichotoma* var. *implexa* obtained from the Northern Adriatic Sea [73]. Indicol (**189**), indcarol acetate (**190**), isolinearol (**191**), and linearol (**192**) (Figure 8) are secodolastane diterpenoids found in *D. dichotoma* var. *indica* from the Arabian Sea [74]. The first total synthesis of racemic isolinearol (**191**) was accomplished from commercially available 2-methyl-1,3-cyclohexanedione in a total of 22 steps [75].

Dictinol (**193**), dictindiol (**194**), and dictintriol (**195**) (Figure 8) are dolastane diterpenoids that have been isolated from the acetone extract of *D*. *dichotoma* var. indica. [76]. Dictyotriol A (**196**) and dictyotriol B (**197**) were detected in *D. dichotoma* var. *indica* collected from the Yellow Sea [77].

Fucoxanthin (**198**) was extracted from *D. dichotoma var. indica*, collected from Qeshm Island, Persian Gulf. In a 24 h treatment at a concentration of 50 µg/mL, it displayed effective anticancer activity against the breast cancer cell line, without toxic effects to the normal cells [78,79].

Palladium oxide nanoparticles using the extract of *D. dichotoma* var. *indica* collected from the Oman Sea coasts in Chabahar, Iran, have a spherical shape with an average particle diameter of 19 nm [80]. Polyshaped gold nanoparticles derived from an extract of leaves from *D. dichotoma* of size 8 ± 21 nm were synthesized. Antibacterial activities were observed by the agar well diffusion method for the action of streptomycin and gentamycin and their formulation with biocapped gold nanoparticles. The profound efficacies were supported by the increase in fold area of inhibition against the tested bacteria [81].

Amijiol (**187**), isoamijiol (**199**), and 14-deoxyamijiol (**200**) were found in the methanolic extract of *D. dichotoma* var. *linearis*, collected from Japan [71,82]. Amijitrienol (**201**) and 14-deoxyisoamijiol (**202**) were detected in the methanolic extract of *D. dichotoma* var. *linearis* from Japan [83].

4-Acetoxy-9,14-dihydroxydolastadiene (**203**), 14-hydroxydolasta-1(15),7,9-triene (**204**), 4,9,14-trihydroxydolasta-1(15),7-diene (**205**), 4,7,14-trihydroxydolasta-1(15),8-diene (**206**), and 4,6-diacetoxy-14-hydroxydolastadiene (**207**), tricyclic diterpenes, were identified in the methanolic extract of *D. dichotoma* var. *linearis*, collected from Honduras Bay Islands. Crude extracts of *D. dichotoma* var. *linearis* and *D. dichotoma* var. *divaricata* showed toxicity to goldfish at 400 µg/mL (death in 90 min) [84].

Isopachydictyolal (**208**) and 4α-acetyldictyodial (**209**) were detected in the methanolic extract of *D. dichotoma* var. *linearis*. The antiviral activity of these metabolites isolated in adequate amounts was evaluated in laboratory assays against *Herpes simplex* virus I (HSV I) and poliomyelitis virus I, using Vero cells as the hosts [85].

2-Methyl-6-[(2S,3αS,4R,5S,7R,8αR)-2,4,7-trihydroxy-3,8-dimethylidenedecahydroazulen-5-yl]hept-2-ene-4,5-diyl diacetate (**210**), 6-methyl-2-[(2*S*,3α*S*,4*R*,5*S*,7*R*,8α*R*)-2,4,7-trihydroxy-3,8-dimethylidenedecahydroazulen-5-yl]hept-5-en-3-yl acetate (**211**), 2-methyl-6-[(3α*R*,4*R*,5*S*,7*R*,8α*R*)-3,4,7-trihydroxy-3-methyl-8-methylidene-3,3α,4,5,6,7,8,8α-octahydroazulen-5-yl]hept-2-ene-4,5-diyl diacetate (**212**), 6-[(3α*R*,4*R*,5*S*,8α*R*)-3,4-dihydroxy-3-methyl-8-methylidene-3,3α,4,5,6,7,8,8α-octahydroazulen-5-yl]-2-methylhept-2-en-4-yl acetate (**213**), and 2-[(3α*S*,4*R*,5*S*,7*R*,8α*R*)-4,7-dihydroxy-3-methyl-8-methylidene-1,3α,4,5,6,7,8,8α-octahydroazulen-5-yl]-5-hydroxy-6-methylhept-6-en-3-yl acetate (**214**) are highly oxidized hydroazulenoid diterpenes from *D. dichotoma* var. *volubilis* [86].

Dictyol G acetate (**11**), 6-[(3α*S*,4*R*,5*S*,7*R*,8α*R*)-4-(acetyloxy)-7-hydroxy-3-methyl-8-methylidene-1,3α,4,5,6,7,8,8α-octahydroazulen-5-yl]-2-methylhept-2-ene-4,5-diyl diacetate (**215**), 6-[(3α*S*,4*R*,5*S*,7*R*,8α*R*)-4,7-dihydroxy-3-methyl-8-methylidene-1,3α,4,5,6,7,8,8α-octahydroazulen-5-yl]-2-methylhept-2-ene-4,5-diyl diacetate (**216**), (4E)-2-[(3α*S*,4*R*,5*S*,7*R*,8α*R*)-4,7-dihydroxy-3-methyl-8-methylidene-1,3α,4,5,6,7,8,8α-octahydroazulen-5-yl]-6-hydroxy-6-methylhept-4-en-3-yl acetate (**217**), 6-methyl-2-[(3α*R*,4*R*,5*S*,7*R*,8α*R*)-3,4,7-trihydroxy-3-methyl-8-methylidene-3,3α,4,5,6,7,8,8α-octahydroazulen-5-yl]hept-5-en-3-yl acetate (**218**) and dilophol (**219**) are highly oxidized hydroazulenoid diterpenes from *D. dichotoma* var. *volubilis* collected from Geoffrey Bay (Australia) (Figure 8) [87].

The influence of temperature (20, 40, and 60 °C) and of the extraction solvents (water, ethanol) on the ultrasound-assisted extraction of phenolics from the Adriatic *D. dichotoma* was recorded. The extracts were analyzed for major phenolic sub-groups (total phenolics, flavonoids, and tannins), while the individual phenolics were detected by HPLC. The antioxidant activities were evaluated using three methods: ferric reducing/antioxidant power (FRAP), scavenging of the stabile 2,2-diphenyl-1-picrylhydrazyl (DPPH) radical, and the oxygen radical antioxidant capacity (ORAC). The total phenolics, flavonoids, and tannins were obtained using an alcoholic solvent, while a general conclusion about the applied temperature was not established. These extracts also showed good antioxidant activity, with high reducing capacity (690–792 mM TE) and ORAC values (38.7–40.8 mM TE in 400-fold diluted extracts). The PCA pointed out the significant influence of flavonoids and tannins on the investigated properties. The results of this investigation could be interesting for future studies dealing with the application of *D. dichotoma* in foods, cosmetics, and pharmaceuticals [88]. The aqueous and ethanolic extracts of the *D. dichotoma*, collected from Hurghada, the Red Sea coast of Egypt, exert an alleviation of salt stress on the germination of rice seeds at concentrations of 0, 5, 10, 20, and 50 g/L. The % germination of rice increased from 84% in non-treated seeds to 100% when treated with 20 g/L. The lower recovery of salt-treated seeds compared with the control seed germination suggests that rice suffered from the toxic ion effect of salinity on embryo rather than from the osmotic effect. Extracts of *D. dichotoma* can enhance and alleviate salinity stress on rice seed germination [89].

### 2.9. Dictyota dumosa Børgesen 1935

Several phytoconstituents were detected in the extracts of *D.* *dumosa*, *D. dichotoma* var. *intricata*, and *D. dichotoma* var. *indica*, collected from the Gulf of Mannar (India), where phenols, terpenoids, and cardiac glycosides were the major constituents. Antibacterial activity was tested against *K. pneumoniae*, *S. aureus*, *P. aeruginosa*, and *B. subtilis*, while antifungal activity was tested against *A. niger*, *A. fumigatus, A. terreus, F. oxysporum*, and *C. albicans*. Antibacterial activity was more promising in all the extracts of seaweed than antifungal activity, with the highest zone of inhibition of 17 mm shown by a methanolic extract of *D. dumosa* against *K. pneumonia* followed by 15 mm exerted by a chloroform extract of *D. dichotoma* var. *indica* against *B. subtilis* [90]. Fatty-acid analyses of *D. dichotoma*, *D. dumosa*, *D. hauckiana*, *D. dichotoma* var. *indica*, and *D. maxima*, found in Karachi coastal waters, evidenced the occurrence of ten saturated and four unsaturated fatty acids. Only palmitic acid occurred in all *Dictyota* species, while eicosatrienoic acid displayed a more selective distribution in *D. indica* [91].

### 2.10. Dictyota fasciola (Roth) J.V. Lamouroux 1809

**Figure 9:**
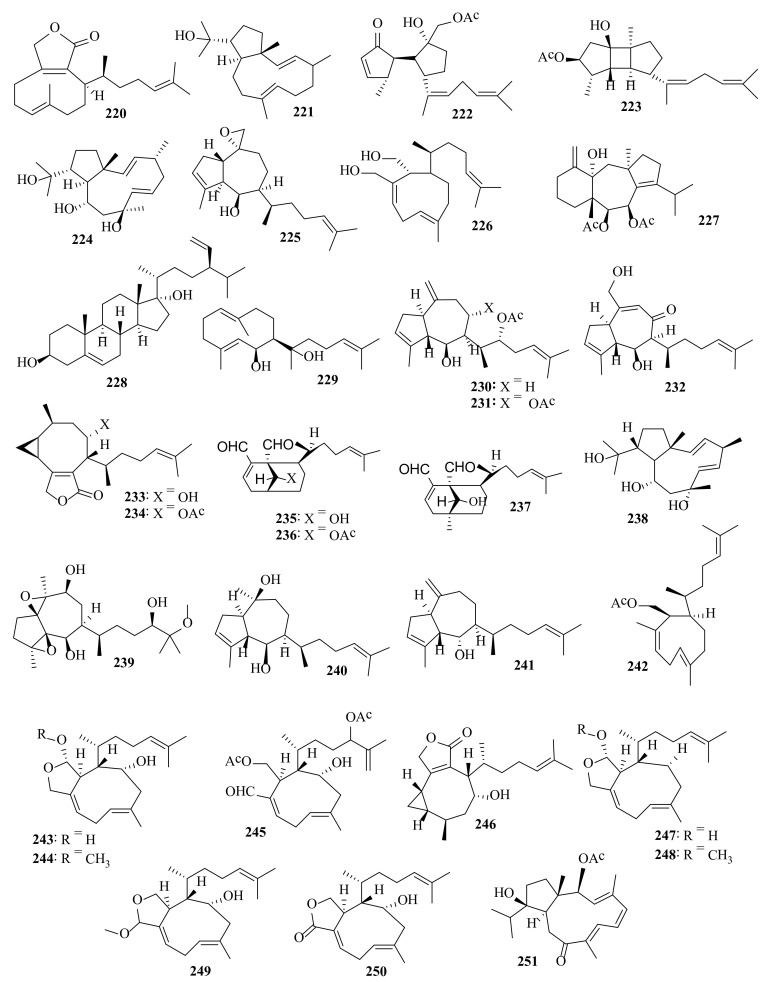
Chemical structures of compounds **220**–**251**. Diterpenes **220**–**221** were isolated from *D. fasciola*, diterpenes **222**–**223** were isolated from *D. fenestrate*, diterpene **224** was isolated from *D. friabilis*, diterpenes **225**–**227** were isolated from *D. furcellata*, sterol **228** was isolated from *D. hauckiana*, diterpenes **229**–**239** were isolated from *D. menstrualis*, diterpenes **240**–**242** were isolated from *D. pinnatifida*, and diterpenes **243**–**251** were isolated from *D. plectens.* The configuration of all stereocenters in each compound is shown as given in the literature.

The dichloromethane and dichloromethane/methanol extracts of *D. fasciola* showed noteworthy growth inhibition of marine bacteria and microalgae. Dictyol C (**24**), sanadaol (**36**), acetyldictyolal (**37**), dictyol E (**112**), neodictyolactone (**220**), and 18-hydroxy-2,7-dolabelladiene (**221**) (Figure 9) were found in the crude extracts of *D. fasciola* collected from northern Tunisia [92].

### 2.11. Dictyota fenestrata J. Agardh 1894

Spatane-derived diterpenoids, secospatane (**222**) and 2-acetoxyspata-13(15),17-dien-10-ol (**223**) were identified in the Australian species *D. fenestrata* (Figure 9) [93].

### 2.12. Dictyota friabilis Setchell 1926

Dolabelladienetriol (**224**), which was detected in *D. friabilis*, collected from Brazil, had a clear effect on HIV-1, inhibiting its replication in cell culture. Pretreatment with dolabelladienetriol (**224**) for 2 h or 1–5 days showed an inhibitory effect ranging from 60 to 90% in peripheral blood mononuclear cells (PBMCs) and macrophages infected with HIV-1, respectively. Dolabelladienetriol (**224**), after being subjected to the experimental model of ex vivo cervical mucosa, did not show any toxicity in concentrations between 14.4 and 0.15 μM. The protective effect of dolabelladienetriol (**224**) in an explant model in the uterine cervix was evaluated. The results show a curve of dose-dependent inhibition upon treatment with different concentrations of the compound in the presence of HIV-1 with 20–99% in concentrations of 0.15 and 14.4 μM, respectively (Figure 9) [94].

### 2.13. Dictyota flabellata (Collins) Setchell and N.L. Gardner 1924

Pachydictyol-A epoxide (**225**) was detected in *D. flabellata*, collected at Sandy Beach, Puerto Peñasco, Sonora Mexico [95]. Dictyodial (**26**), dictyolactone (**75**), and dictyodiol (**226**) (Figure 9) were found in *D. crenulata* and *D. flabellata* collected from Hawaii [96]. The total polyphenol content (70 mg/100 g fresh weight) and the vitamin C content (3 mg/g dry weight) in *D. flabellata* collected from Bahia Magdalena, Baja California Sur, Mexico, were determined [97].

### 2.14. Dictyota furcellata (C. Agardh) Greville 1830

In the Australian species *D. furcellata*, the dolastane diterpene derivative 6,7-diacetoxydolasta-1(15),8-dien-14-ol (**227**) (Figure 9) was identified [98].

### 2.15. Dictyota guineënsis (Kützing) P. Crouan and H. Crouan 1878

In the crude extract of *D. guineënsis*, collected from Itamaracá Island (Brazil), five diterpenes were identified: dictyol E (**112**) (which was the most abundant diterpene), pachydictyol A (**14**), dictyoxide (**15**), isopachydictyol A (**17**), and dictyotadiol (**118**) [99].

### 2.16. Dictyota hauckiana Nizamuddin 1975

Hauckiosterol (**228**) was found in *D. hauckiana*, collected from the beaches of Hawkes Bay (New Zealand) [100]. Ethanolic extracts of *D. dichotoma* var. *velutricata*, *D. dichotoma* var. *indica* and *D. hauckiana*, occurring at Karachi Coast were screened for their cytotoxic activity using brine shrimp lethality for larvae (nauplii). The ethanolic extract of this species exhibited significant cytotoxicity (LC_50_ < 1000 μg) on brine shrimps. *D. dichotoma* var. *indica* showed the highest cytotoxic activity (LC_50_ = 141 µg), while *D. hauckiana* was found to be moderately effective on brine shrimp nauplii (LC_50_ = 524 µg). *D. dichotoma* var. velutricata were found to be less cytotoxic (LC_50_ = 812 µg) [101].

### 2.17. Dictyota masonii Setchell and N.L. Gardner 1930

The monocyclic diterpenoid hydroxydilophol (**229**) (Figure 9) was detected in the brown alga *D. masonii* collected from Isla (Spain) [102].

### 2.18. Dictyota menstrualis (Hoyt) Schnetter, Hörning and Weber-Peukert 1987

Dictyol C (**24**), acetoxycrenulide (**33**), dictyotin A (**69**), dictyol K (**230**), and dictyol M (**231**), dictyol N (**232**), isoacetoxycrenulatin (**233**), and 4-hidroxycrenulide (**234**) (Figure 9) were found in the Brazilian alga *D. menstrualis*. Anti-inflammatory, antimicrobial, and cytotoxic assays were performed with the isolated compounds, but only the anti-inflammatory activity was particularly prominent. The cell viability effects of the detected compounds on the murine macrophage cell line RAW 264.7 showed IC_50_ values from 1.12 to 2.53 μM, and the inhibitory activity against the nitric oxide production over the lipopolysaccharide-stimulated RAW 264.7 cell line was observed for all compounds, with IC_50_ values ranging from 0.12 to 0.23 mM [103].

The antiviral effect of the CH_2_Cl_2_/MeOH-soluble fraction from the Brazilian *D. menstrualis* on HIV-1 replication was evaluated in vitro. The antiretroviral activity was attributed to two diterpenes: (6*R*)-6-hydroxydichotoma-3,14-diene-1,17-dial (**235**), and (6*R*)-6-acetoxidichotoma-3,14-diene-1,17-dial (**236**). These dialdehydes affected an early step of the HIV-1 virus replicative cycle in a dose-dependent manner [104]. Moreover, the effects of three diterpenes, pachydictyol A (**14**), isopachydictyol A (**17**), and dichotomanol (**237**), on platelet aggregation and plasma coagulation were studied. Dichotomanol (**237**) inhibited ADP- or collagen-induced aggregation of platelet-rich plasma (PRP) but failed to inhibit washed platelets (WP). Pachydictyol A (**14**) and isopachydictyol A (**17**), by contrast, failed to inhibit the aggregation of PRP, but impaired WP aggregation induced by collagen or thrombin and inhibited coagulation, as analyzed by the prothrombin time and activated partial thromboplastin time and on commercial fibrinogen. Moreover, some of the isolated diterpenes inhibited the catalytic activity of thrombin [105].

(6*R*)-6-Hydroxydichotoma-4,14-diene-1,17-dial (**235**) and 8,10,18-trihydroxy-2,6-dolabelladiene (**238**) were discovered in Brazilian *D. menstrualis*. They were found to inhibit HSV-1 infection in Vero cells. Compounds **235** and **238** (Figure 9) inhibited HSV-1 replication in a dose-dependent manner (EC_50_ = 90 and 5.10 µM, respectively) [106].

Methanolic extracts of *D. ciliolata* (MEDC) and *D. menstrualis* (MED), collected from the Northeast of Brazil, were assessed as apoptosis-inducing agents on human cervical adenocarcinoma (HeLa) cells. All extracts showed different levels of cytotoxicity against the cells. In addition, MEDC and MEDM also inhibited SiHa (cervix carcinoma) cell proliferation. Studies with these two extracts showed that HeLa cells exposed to MEDM and MEDC exhibited morphological and biochemical changes that characterize apoptosis as shown by loss of cell viability, chromatin condensation, phosphatidylserine externalization, and sub-G1 cell cycle phase accumulation, and MEDC also induces cell cycle arrest in cell cycle phase S [107].

Extracted sulfated polysaccharides from Brazilian *D. menstrualis*, followed by separation into five fractions by sequential acetone precipitation, demonstrated that all fractions were composed mainly of fucose, xylose, galactose, uronic acid, and inorganic sulfate. The anticoagulant activity of these heterofucans was determined by activated partial thromboplastin time (APTT) using citrate normal human plasma. Only the fucans F1.0v and F1.5v showed anticoagulant activity. To prolong the coagulation time to double the baseline value in the APTT, the required concentration of fucan F1.0v (20 µg/mL) was only 4.88-fold higher than that of the low-molecular-weight heparin Clexane^®^ (4.1 µg/mL), whereas 80 µg/mL fucan 1.5 was needed to obtain the same effect. For both fucans, the effect was abolished by desulfation [108].

A heterofucan (F2.0v) from *D. menstrualis*, collected from Búzios Beach, Rio Grande do Norte State (Brazil), was evaluated as an antinociceptive and anti-inflammatory agent. F2.0v (20.0 mg/kg) inhibited 100% of leukocyte migration into the peritoneal cavity after chemical stimulation. However, F2.0v did not alter the expression of interleukin-1 beta (IL-1β) and interleukin-6 (IL-6), as well as tumor necrosis factor-alpha (TNF-α). F2.0v (20.0 mg/kg) had peripheral antinociceptive activity with potency like dipyrone. On the other hand, it did not affect pain response on the hot-plate test. Confocal microscopy analysis and flow cytometry showed that F2.0v binds to the surface of leucocytes, which leads us to suggest that the mechanisms of action of anti-inflammatory and antinociceptive F2.0v are related to its ability to inhibit the migration of leukocytes to the site of tissue injury. In summary, the data show that the F2.0v compound may have a great potential as an antinociceptive and anti-inflammatory agent [109]. The crude extract of Brazilian *D. menstrualis* and its derived fractions were analyzed for their antiviral potential, alone and in combination with ribavirin at 20 μg/mL. The fractions that were rich in cyclic diterpenes with aldehyde groupings, inhibited *Zika virus* replication by >74%, with inhibition behaving in a dose-dependent manner, with EC_50_ values of 2.80 (F-6) and 0.81 (FAc-2) μg/mL. Regarding the mechanism of action, FAc-2 had a strong virucidal potential, and F-6 inhibited viral adsorption. Associating FAc-2 with ribavirin at suboptimal dosages produced a strong synergistic effect that completely inhibited viral replication [110].

### 2.19. Dictyota mertensii (C. Martius) Kützing 1859

Fucan-coated silver nanoparticles (FN) from *D. mertensii* inhibited the proliferation of the melanoma tumor cell line B16F10 (60%). In addition, they had immunomodulatory properties: They caused an up to 7000-fold increase in the release of nitric oxide and cytokines (IL-10; IL-6 and TNF-α). In addition, the FN showed an inhibitory effect against both, Gram-positive and Gram-negative bacteria (MIC = 50 µg/mL). Overall, the data showed that FN is a nanoparticle with the potential to be used as an antitumor, immunomodulatory, and antibacterial agent [111]. Pachydictyol A (**14**), isopachydictyol A (**17**), dictyol B acetate (**18**), dictyol C (**24**), and dictyol B (**77**) were isolated from *D. mertensii* [112].

### 2.20. Dictyota pinnatifida Kützing 1859

In *D. pinnatifida*, collected from the Colombian Caribbean, dictyoxepin (**4**), dictyol L (**239**), 6-epipachydictyol A (**240**), 6-epidictyol C (**241**), and 18-acetoxy-xenianol (**242**) were identified (Figure 9). Dictyol L (**239**) displayed strong inhibition of *P. aeruginosa* biofilms, better than kojic acid, which was used as the control. Dictyoxepin (**4**) showed mild inhibition of *E. coli* biofilms [113].

### 2.21. Dictyota plectens (Allender and Kraft) Kraft 2009

**Figure 10:**
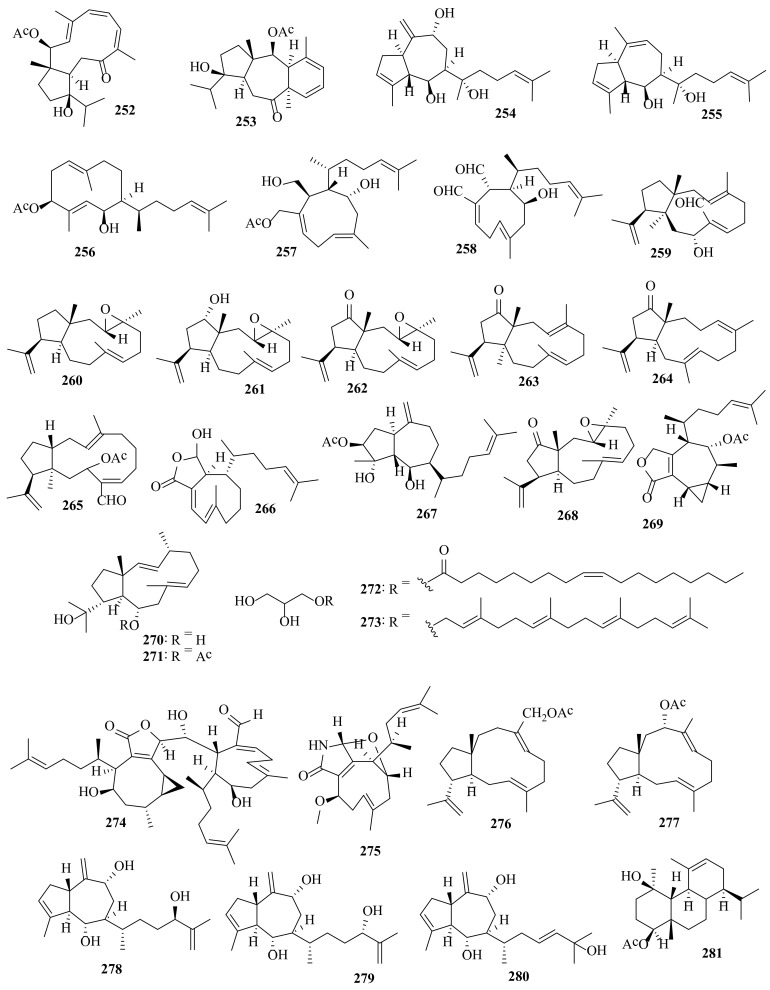
Chemical structures of compounds **252**–**281**. Diterpenes **252**–**257** were isolated from *D. plectens*, diterpene **258** was isolated from *D. spinulosa*, diterpenes **259**–**227** were isolated from *D. spiralis*, and diterpenes **265**–**271** and **274**–**281** and glycerol derivatives **272**–**273** were isolated from *Dictyota* sp. The configuration of all stereocenters in each compound is shown as given in the literature.

**Figure 11:**
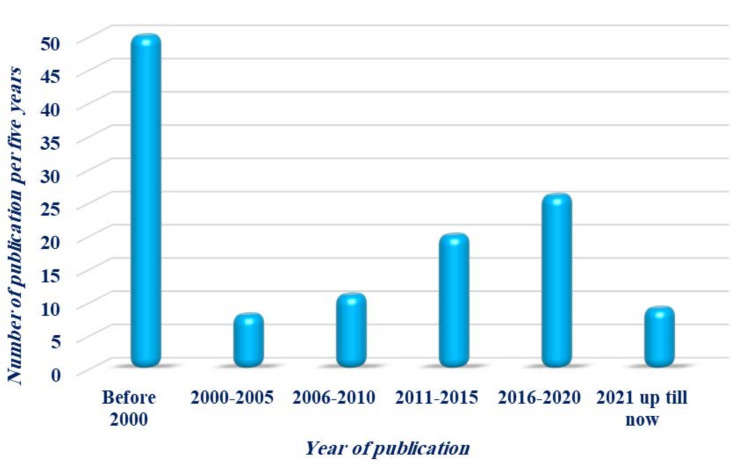
The rate of publication on the genus *Dictyota*.

4α-Hydroxyisodictyohemiacetal (**243**), 4α-hydroxyisodictyoacetal (**244**), 13,18-diacetoxy-4-hydroxyisodictyo-19-al (**245**), 4α-hydroxypachylactone (**246**), isodictyohemiacetal (**247**), isodictyoacetal (**248**), (2*S*,3*S*,4*R*,10*R*,19*R*)-19-deoxo-4-hydroxy-19-methoxydictyolactone (**249**), and 4-hydroxydictyolactone (**250**) (Figure 9) were found in the Chinese alga *D. plectens*. All compounds were evaluated for their antiviral activities against human immunodeficiency virus type 1 (HIV-1) replication activity. 4α-Hydroxyisodictyohemiacetal (**243**) and isodictyoacetal (**248**) were active against the replication of wild-type HIV-1 virus with inhibitory concentration 50% (IC_50_) of 28.1 and 25.4 µM, while the other compounds were inactive at a concentration of 30.0 µM. Hydroxypachylactone (**246**) showed specific inhibition (66.8% at 30.0 µM) against hemagglutinin (HA)-mediated  highly pathogenic H5N1 infection utilizing an HIV-based pseudotyping system. 4α-Hydroxypachylactone (**246**) and 4-hydroxydictyolactone (**250**) effectively inhibited lipopolysaccharide (LPS)-induced nitric oxide (NO) production in mouse peritoneal macrophages (PEMΦ), with inhibition rates of 76.0% and 53.2%, respectively, at 10.0 µM, whereas the other compounds showed only weak activities [114]. (1*S*,2*S*,3E,5Z,7E,11*R*,12*R*)-2-acetoxy-12-hydroxydolabella-3,5,7-trien-9-one (**251**) (Figure 9), (1*S*,2*S*,3E,5Z,7Z,11*R*,12*R*)-2-acetoxy-12-hydroxydolabella-3,5,7-trien-9-one (**252**), (1*S*,2*S*,3*S*,4Z,6Z,8*R*,11*R*,12*R*)-2-acetoxy-12-hydroxydolasta -4,6-dien-9-one (**253**), 9α-hydroxydictyol E (**254**), isodictyol E (**255**), 3β-acetoxydilophol (**256**), and 19-acetyl-4-hydroxydictyodiol (**257**) (Figure 10) were found in *D. plectens* collected from the South China Sea [115].

### 2.22. Dictyota pulchella Hörnig and Schnetter 1988

The cardiovascular effects elicited by *D. pulchella* were investigated using in vitro and in vivo experiments. In normotensive conscious rats, CH_2_Cl_2_/MeOH extract (CME, 5, 10, 20, and 40 mg/kg) from *D. pulchella* produced dose-dependent hypotension (−4 ± 1; −8 ± 2; −53 ± 8; and −63 ± 3 mmHg) and bradycardia (−8 ± 6; −17 ± 11; −257 ± 36; and −285 ± 27 b.p.m.). In addition, the CME and hexane/EtOAc phases (HEP) (0.01–300 µg/mL) from *D. pulchella* induced a concentration-dependent relaxation in mesenteric artery rings pre-contracted with phenylephrine (Phe, 1 µM). The vasorelaxant effect was not modified by the removal of the vascular endothelium or by pre-incubation with KCl (20 mM), tetraethylammonium (TEA, 3 mM), or thromboxane A2 agonist U-46619 (100 nM). Furthermore, CME and HEP reversed CaCl_2_-induced vascular contractions. These results suggest that both CME and HEP acted on the voltage-operated calcium channel to produce vasorelaxation. In addition, CME-induced vasodilatation occurred after the vessels had been pre-contracted with an L-type Ca^2+^ channel agonist (Bay K 8644, 200 nM). Taken together, these data show that CME induced hypotension and bradycardia in vivo and that both CME and HEP induced endothelium-independent vasodilatation in vitro that seemed to involve the inhibition of the Ca^2+^ influx through the blockade of voltage-operated calcium channels [116]. Despite these interesting activities, no pure compounds have so far been isolated from this seaweed.

### 2.23. Dictyota sandvicensis Sonder 1859

*D. sandvicensis* is an edible Hawaiian seaweed. Its contents (%) of water (relative to the total fresh weight), total ash, total soluble protein, total soluble carbohydrates and crude lipids (relative to the total dry weight) were measured (86.4 ± 0.3, 28.9 ± 0.1, 6.4 ± 0.6, 6.7 ± 0.4, and 20.2 ± 0.1, respectively) [117]. No pure compounds have so far been isolated from this species.

### 2.24. Dictyota spinulosa J.D. Hooker and Arnott 1838

In *D. spinulosa*, collected from Kin, Okinawa (Japan), hydroxydictyodial (**258**) (Figure 10) was found. In feeding tests with Mozambique tilapia (*Oreochromis mossambicus*), it acted as an antifeedant diterpene [118],

### 2.25. Dictyota spiralis Montagne 1846

Spiralyde A (**259**), (1*R*,3*S*,4*S*,7*E*,11*S*,12*S*)-3,4-epoxy-7,18-dolabelladiene (**260**), (1*R*,3*S*,4*S*,7*E*,11*S*,12*S*,14*S*)-3,4-epoxy-14-hydroxy-7,18-dolabelladiene (**261**), (1*R*,3*S*,4*S*,7*E*,11*S*,12*S*)-3,4-epoxy-14-oxo-7,18-dolabelladiene (**262**), (1*R*,3E,7*E*,11*S*,12*S*)-14-oxo-3,7,18-dolabellatriene (**263**), and (1*R*,3Z,7*E*,11*S*,12*S*)-14-oxo-3,7,18-dolabellatriene (**264**) (Figure 10) were detected in *D. spiralis*, collected from the intertidal zone of the northwest coast of Tunisia. The dichloromethane extract of *D. spiralis* exhibited a promising antikinetoplastid capacity, with an IC_50_ of 9.76 ± 0.55 and 8.82 ± 0.98 µg/mL against the promastigote form of *Leishmani amazonensis* and the epimastigote form of *Trypanosoma cruzi*, respectively. Among the compounds tested, spiralyde A (**259**) was the most active agent against *L. amazonensis* and *T. cruzi* [119]. Dictyol E (**112**), dolabellane (**265**), xenicane (**266**), prenylated guaiane (**267**), 3,4-epoxy-14-oxo-7,18-dolabelladiene (**268**), acetoxycrenulide (**269**), 10,18-dihydroxydolabella-2,7-diene (**270**), 10-acetoxy-18-hydroxydolabella-2,7-diene (**271**), 1-*O*-octadecenoylglycerol (**272**), and sn-3-*O*-(geranylgeranyl)glycerol (**273**) (Figure 10) were identified in *Dictyota* sp., collected from French and Algerian Mediterranean coastal sites. Compounds **268**–**269** and **271**–**273** were screened for their potential to prevent the adhesion of three bacterial strains isolated from marine biofilms in comparison with four commercial antifoulants. Compounds **272**–**273** exhibited the strongest anti-adhesion effects, with moderate toxicity [120].

Dictyotadimer A (**274**), a dissymmetric C_40_ bis-diterpene, characterized by a C–C linkage between two different xenicane units, was discovered in the Mediterranean Sea grass *Dictyota* sp. [121]. Joalin (**275**) is the first nitrogen-containing xenicane diterpene detected in *Dictyota* sp., collected from the Senegalese Coast [122]. 16-Acetoxy-1*R*,11*S*,12*R*-dolabella-triene (**276**) and 3*S*-acetoxy-1*R*,11*S*,12*R*-dolabella-triene (**277**) (Figure 10) are minor dolabellane diterpenoid constituents from a *Dictyota* sp. [123]. The three hydroazulenoid diterpenes dictyotriol C (**278**), dictyotriol D (**279**), and dictyotriol E (**280**), along with α-dictalediol monoacetate (**281**) (Figure 10), were identified in *Dictyota* sp*.*, collected from the Canary Islands [124,125]. Pachydictyol C (**111**), Dictyol E (**112**), and 3,4-epoxy-7,18-dolabelladiene have been identified in *D. spiralis* collected along the coast of Tunisia. In vitro evaluation of the trypanocidal activity exhibited IC_50_ values ranging from 13.11 to 35.28 µM for these compounds; in comparison to benznidazole, which was active with an IC_50_ of 6.95 µM. Regarding cytotoxicity, CC_50_ values were found to range from 36.96 µM for pachydictyol C (**111**) to 69.98 µM for 3,4-epoxy-7,18-dolabelladiene compared to miltefosine and benznidazole [126]. Diterpenes such as dictyol C (**24**), (8*R*,11*R*)-8,11-diacetoxypachydictyol A, (8R*,11R*)-6-*O*-acetyl-8-acetoxy-11-hydroxypachydictyol A, (8R*,11S*)-8-acetoxy-11-hydroxypachydictyol A, and (8R*,11S*)-6-*O*-acetyl-8,11-dihydroxypachydictyol A, and a secohydroazulene derivative, named 7*Z*-7,8-seco-7,11-didehydro-8- acetoxypachydictyol A, were detected in *Dictyota* sp. collected from the South China Sea. The potent antioxidant effects against H_2_O_2_-induced oxidative damage in neuron-like PC12 cells took place at a low concentration of 2 μM. The antioxidant property of dictyol C (**24**) was associated with an activation of the Nrf2/ARE signaling pathway; it also showed neuroprotective effects against cerebral ischemia-reperfusion injury (CIRI) in a rat model of transient middle cerebral artery occlusion. As such, hydroazulene diterpenes could serve as lead structures for the development of novel neuroprotective agents against CIRI [127]

## 3. Conclusions and Future Directions

The genus *Dictyota* is one of the most important brown algae in the coastal zones because of its biomass and wide distribution in tropical to temperate seas. Despite the folk uses of brown algae, particularly of *Dictyota*, all over the world, the knowledge about its phytopharmacological potentials is still underexplored. Several species, such as *D. divaricata*, *D. implexa*, *D. indica*, *D. linearis*, and *D. volubilis*, have recently been considered the holotype of the genus *Dictyota*. Analysis of the reported data regarding its phytoconstituents has implied some remarkable gaps in the wholeness of our perception of their attribution to the reported biological activities and health outcomes of *Dictyota*, besides the roles performed by each of these metabolites. Moreover, existing biological research on several *Dictyota* species is particularly limited to the investigation of their total extracts or fractions, while the isolated components have so far received much less interest. Hence, further studies should comprehensively be done to discover the metabolites and their actual contribution to the medicinal properties of *Dictyota*, in addition to the implied mechanisms and their possible synergistic interactions, which would permit a sensible application of the genus *Dictyota* in modern phytotherapy. From a medicinal point of view, the antiproliferative potential of the genus *Dictyota* represents an indispensable future research theme. Many studies have extensively approached its cytotoxic properties. However, despite its reported cytotoxicity, the mechanism of action should be considered in future research to best appreciate this potential. Several dermal applications of the genus *Dictyota* were reported, such as hypopigmentation and anti-alopecia, yet so far without an application of these effects in cosmetic production. Species of the genus *Dictyota* were found to be cardioprotective and neuroprotective; however, further investigations are required for a full investigation of these effects and the effects on other body organs.

The antimicrobial potential is another considerable challenge because the current reported antimicrobial data are mostly restricted to crude extracts and fractions of *Dictyota* species. Although some of them exhibited high, broad-spectrum antimicrobial activities, a further detailed investigation of the antimicrobial properties of *Dictyota* following standardized protocols, involving those of the purified metabolites, is still missing and, hence, highly recommended. The investigation of the bioactive metabolites obtained from the genus *Dictyota* should be combined with more in-depth structure–activity relationship studies, and with the elucidation of their possible mechanisms of action. Elucidating the cellular and molecular features of the biological activities of *Dictyota* metabolites will be of pronounced value in the discovery and development of new bioactive compounds. Moreover, artificial analogs of bioactive metabolites of the genus *Dictyota* should be designed, synthesized, and tested, focusing on improving the efficacy and safety, and on increasing its economic production for nutrition with its further investigation as a natural source of new drugs.

Studies of nanotechnology could permit a safe and effective application of *Dictyota* species in modern phytotherapy. Many industrial applications as a source of biofuels and agricultural applications as a source of growth-promoting agents of this species are imaginable, but have not been sufficiently studied yet. The publication rate regarding *Dictyota* has been rising vigorously within the past five years (Figure 11), illustrating the rapidly growing interest in this intriguing genus.

**Figure 12:**
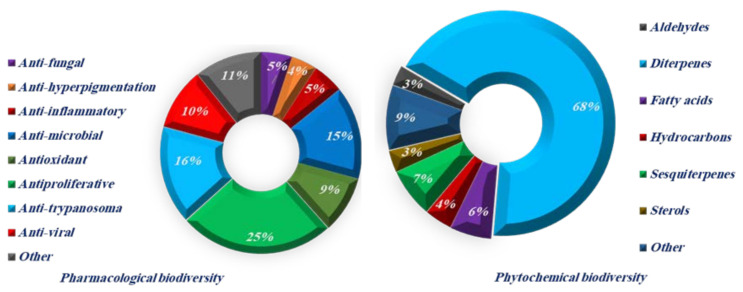
Pharmacological and phytochemical biodiversity of *Dictyota*.

So far, 25 species names of *Dictyota*, representing 24.5% of the current species, have been accepted taxonomically and investigated for their chemical and pharmacological activities (Figure 12), of which the most studied species as a possible drug source is *D. dichotoma*.

Several bioactive compounds with most diverse chemical structures have been reported, including diterpenes sesquiterpenes, carotenoids, hydrocarbons, terpenes, sterols, and sulfated polysaccharides (Figure 2, Figure 3, Figure 4, Figure 5, Figure 6, Figure 7, Figure 8, Figure 9 and Figure 10, Tables S1 and S2). These isolated compounds and/or extracts displayed diverse biological activities such as antiproliferative, antimicrobial, antiviral, antioxidant, anti-inflammatory, and anti-hyperpigmentation activities. In addition, fucoxanthin displayed antiproliferative activities towards different cancer cell lines. Moreover, the high polyphenolic content of some *Dictyota* sp. mainly reflects their antioxidant and antimicrobial activities. Finally, several *Dictyota* sp. extracts exhibited antiviral activities against HSV and HIV. The data compiled in the current review collectively highlight the potential of the genus *Dictyota* as a promising candidate for the development of alternative medicinal agents and, in the same context, the effective utilization of this alga as a functional-food ingredient, especially considering its known nutritional value, is also warranted.

## Supplementary Materials

The following supporting information can be downloaded, Table S1: Pharmacological potential of compounds isolated from the genus Dictyota; Table S2: Biological assays related to therapeutic potential of *Dictyota* extracts.
