# Supplementary material for: The Biodiversity of the Genus Dictyota: Phytochemical and Pharmacological Natural Products Prospectives
Source: Molecules. 2022 Jan 20;27(3):672. doi: 10.3390/molecules27030672 (PMC8838102; doi:10.3390/molecules27030672)
Supplement: Supplementary file 1 [file molecules-27-00672-s001.zip › molecules-1541480-supplementary.pdf]

Supplementary Materials

Table S1: Pharmacological potential of compounds isolated from the genus *Dictyota*

No.	Name	Species	Class	Activity	References
**1**	Acutilol A	*D. acutiloba*	Diterpene	Chemical defense	[11]
**2**	Acutilol A acetate	*D. acutiloba*	Diterpene	Chemical defense	[11]
**3**	Acutilol B	*D. acutiloba*	Diterpene	Chemical defense	[11]
**4**	Dictyoxepin	*D. acutiloba*<br>*D. pinnatifida*	Diterpene	Antimicrobial	[11, 113]
**5**	Dictyolene	*D. acutiloba*	Diterpene	-	[11]
**6**	9-Hydroxydolabelladien-6-one	*D. bartayresiana*	Diterpene	-	[13]
**7**	5-Acetoxy-12-hydroxydolabell-3,7*Z*-dienone	*D. bartayresiana*	Diterpene	-	[13]
**8**	9-Acetoxydolabellatrien-16-al	*D. bartayresiana*	Diterpene	-	[13]
**9**	5-Acetoxy-12-hydroxydolabell-3,7*E*-dienone	*D. bartayresiana*	Diterpene	-	[13]
**10**	Trihydroxydolasta-2-en-6-one	*D. bartayresiana*	Diterpene	-	[13]
**11**	Dictyol G acetate	*D. binghamiae,*<br>*D. dichotoma*	Diterpene	-	[18, 87]
**12**	Dictyoxide A	*D. binghamiae*	Diterpene	-	[18]
**13**	Dictyotriol A diacetate	*D. binghamiae*	Diterpene	-	[18]
**14**	Pachydictyol A	*D. binghamiae,*<br>*D. ciliolata,*<br>*D. dichotoma,*<br>*D. guineensis,*<br>*D. menstrualis,*<br>*D. mertensii*	Diterpene	Antiproliferative	[18, 21, 22, 25, 33, 61, 99, 105, 112]
**15**	Dictyoxid	*D. binghamiae,*<br>*D. guineensi*	Diterpene	-	[18, 99]
**16**	Diacetal	*D. binghamiae*	Diterpene	-	[18]
**17**	Isopachydictyol	*D. caribaea,*<br>*D. dichotoma,*<br>*D. guineensis,*<br>*D. menstrualis,*<br>*D. mertensii*	Diterpene	Antimicrobial	[4, 21, 22, 33, 99, 105, 112]
**18**	Dictyol B acetate	*D. caribaea,*<br>*D. ciliolata,*<br>*D. dichotoma,*<br>*D. mertensi*	Diterpene	Antiproliferative, Antimicrobial	[21, 22, 25, 62, 112]
**19**	Dichotomanol	*D. caribaea*	Diterpene	-	[21, 22]
**20**	Dichotomanol acetate	*D. caribaea*	Diterpene	-	[21, 22]
**21**	Cycloxenianol acetate	*D. caribaea*	Diterpene	-	[21, 22]
**22**	Ciliolatale	*D. ciliolata*	Diterpene	Antifungal	[23]
**23**	17-Acetoxy-dictyodial	*D. ciliolata*	Diterpene	Antifungal	[23]

**24**	Dictyol C	*D. ciliolata, D. dichotoma, D. fasciola, D. menstrualis, D. mertensii, Dictyota* sp.	Diterpene	Antifungal, Anti-inflammatory Antimicrobial, Antioxidant, Neuroprotective	[23, 33, 72, 92, 103, 112, 127]
**25**	Dictyol H	*D. ciliolata*	Diterpene	Antifungal	[23]
**26**	Dictyodial	*D. ciliolata, D. flabellata*	Diterpene	Antifungal	[23, 33, 96]
**27**	3-*O*-(6′-deoxy-6′-sulfo-α-D-glucopyranosyl)-1-*O*-oleoyl-2-*O*-palmitoylglycerol	*D. ciliolata*	Sulfonoglycolipid	-	[24]
**28**	Dictyospiromide	*D. coriacea*	Alkaloid	Antioxidant	[26]
**29**	1,9-Dihydroxycrenulide	*D. coriacea*	Diterpene	Hypopigmentation	[27]
**30**	Epiloliolide	*D. coriacea*	Diterpene	Hypopigmentation	[27]
**31**	D-Mannitol	*D. coriacea*	Carbohydrate	-	[27]
**32**	4β-Hydroxydictyodial A	*D. crenulata*	Diterpene	-	[30, 33]
**33**	Acetoxycrenulide	*D. crenulata, D. menstrualis*	Diterpene	Anti-inflammatory, Antimicrobial	[31, 54, 103]
**34**	Dictyocrenulol	*D. crenulata*	Diterpene	-	[31]
**35**	β-Crenulal	*D. crenulata*	Diterpene	-	[32]
**36**	Sanadaol	*D. crenulata, D. dichotoma, D. fasciola*	Diterpene	Algicidal	[32, 48, 92]
**37**	Acetyldictyolal	*D. dichotoma, D. fasciola*	Diterpene	-	[43, 92]
**38**	Hydroxyacetyldictyolal	*D. dichotoma*	Diterpene	-	[43]
**39**	Isodictyohemiacetal	*D. dichotoma*	Diterpene	-	[43]
**40**	Dictydiacetal	*D. dichotoma*	Diterpene	-	[43]
**41**	4-Acetoxydictyolactone	*D. dichotoma*	Diterpene	-	[44]
**42**	Dictyotalide A	*D. dichotoma*	Diterpene	-	[44]
**43**	Dictyotalide B	*D. dichotoma*	Diterpene	-	[44]
**44**	Nordictyotalide	*D. dichotoma*	Diterpene	-	[44]
**45**	*ent*-Erogorgiaene	*D. dichotoma*	Diterpene	-	[45]
**46**	1,5-Cyclo-tetrahydroerogorgiaene	*D. dichotoma*	Diterpene	-	[45]
**47**	Acetoxy-hydroxy-dolabella-3,7-dien-9-one	*D. dichotoma*	Diterpene	-	[46]
**48**	3,4-Epoxy-hydroxy-dolabella-7-en-9-one	*D. dichotoma*	Diterpene	-	[46]
**49**	7,8-Epoxy-hydroxy-dolabella-3-en-9-one	*D. dichotoma*	Diterpene	-	[46]
**50**	9-Acetoxydolabella-3,7,12-trien-16-al	*D. dichotoma*	Diterpene	-	[46]

**51**	9-Acetoxydolabellatrien-16-oic acid	*D. dichotoma*	Diterpene	-	[46]
**52**	9-Acetoxydolabella-3,7-dien-12-ol	*D. dichotoma*	Diterpene	-	[46]
**53**	9-Acetoxy-7,8-epoxydolabella-3-en-12-ol	*D. dichotoma*	Diterpene	-	[46]
**54**	9-Hydroxydolest-1,3-dien-6-one	*D. dichotoma*	Diterpene	-	[46]
**55**	4,12-Dihydroxydolabella-2,7Z-dien-9-one	*D. dichotoma*	Diterpene	-	[46]
**56**	4,12-Dihydroxydolabella-2,7E-dien-9-one	*D. dichotoma*	Diterpene	-	[46]
**57**	12-Hydroxydolabella-3,7*E*-dien-9-one	*D. dichotoma*	Diterpene	-	[46]
**58**	12-Hydroxydolabella-3,7Z-dien-9-one	*D. dichotoma*	Diterpene	-	[46]
**59**	4,12-Dihydroxydolabellan-2,6-dien-9-one	*D. dichotoma*	Diterpene	-	[46]
**60**	4,12-Dihydroxydolabellan-2,6-dien-9-one	*D. dichotoma*	Diterpene	-	[46]
**61**	12*Z*-Hydroxydolabella-3,6-dien-9-one	*D. dichotoma*	Diterpene	-	[46]
**62**	12*E*-Hydroxydolabella-3,6-dien-9-one	*D. dichotoma*	Diterpene	-	[46]
**63**	5,6,18-Triacetoxy-hydroxy-dolabelladiene	*D. dichotoma*	Diterpene	-	[4]
**64**	18-Acetoxy-10-hydroxy-2,7-dolabelladiene	*D. dichotoma*	Diterpene	-	[4]
**65**	5-Acetoxy-dihydroxy-2,7-dolabelladiene	*D. dichotoma*	Diterpene	-	[4]
**66**	7,8-Epoxy-3,18-dolabelladiene	*D. dichotoma*	Diterpene	-	[4]
**67**	18-Acetoxy-2,7-dolabelladiene	*D. dichotoma*	Diterpene	-	[4]
**68**	Dictyotatriol A	*D. dichotoma*	Diterpene	-	[4]
**69**	Dictyotin A	*D. dichotoma, D. menstrualis*	Diterpene	Anti-inflammatory, Antimicrobial	[47, 103]
**70**	Dictyotin B	*D. dichotoma*	Diterpene	-	[47]
**71**	Dictyotin C	*D. dichotoma*	Diterpene	-	[47]
**72**	(1*R*,4*S*,4α*R*,8α*R*)-1,6-Dimethyl-4-[(2*R*)-6-methylhept-5-en-2-yl]-1,2,3,4,4α,7,8,8α-octahydronaphthalen-1-yl methyl ether	*D. dichotoma*	Diterpene	-	[47]
**73**	Dictyotin D methyl ether	*D. dichotoma*	Diterpene	-	[47]
**74**	Dictyol J	*D. dichotoma*	Diterpene	Algicidal	[48]
**75**	Dictyolactone	*D. dichotoma, D. flabellata*	Diterpene	Algicidal	[48, 96]

**76**	Dictyol A	*D. dichotoma*	Diterpene	-	[49]
**77**	Dictyol B	*D. dichotoma, D. mertensii*	Diterpene	Antimicrobial	[49]
**78**	Dictyol F	*D. dichotoma*	Diterpene	-	[50]
**79**	Epidictyol F	*D. dichotoma*	Diterpene	-	[50]
**80**	Dihydromethoxy-pachydictyol A	*D. dichotoma*	Diterpene	-	[50]
**81**	14,15-Epoxy-pachydictyol A	*D. dichotoma*	Diterpene	-	[50]
**82**	2,6-Cycloether pachydictyol A	*D. dichotoma*	Diterpene	-	[50]
**83**	9*R*-Hydroxydichotoma-2,14-dien-19,20-diol	*D. dichotoma*	Diterpene	-	[51]
**84**	9*R*-Acetoxydichotoma-2,14-dien-19,20-diol	*D. dichotoma*	Diterpene	-	[51]
**85**	7-Hydroxy-2,6-cycloxenicadien-18,19-diol	*D. dichotoma*	Diterpene	-	[51]
**86**	7-Acetoxy-2,6-cycloxenicadien-18,19-diol	*D. dichotoma*	Diterpene	-	[51]
**87**	Crenulacetal A	*D. dichotoma*	Diterpene	-	[52]
**88**	Crenulacetal B	*D. dichotoma*	Diterpene	-	[52]
**89**	Crenulacetal C	*D. dichotoma*	Diterpene	-	[52]
**90**	Crenulacetal D	*D. dichotoma*	Diterpene	-	[52]
**91**	Dichotone	*D. dichotoma*	Diterpene	-	[53]
**92**	Dichotodione	*D. dichotoma*	Diterpene	-	[53]
**93**	Dictyohydroperoxide	*D. dichotoma*	Diterpene	-	[54]
**94**	Hydroperoxyacetoxycrenulide	*D. dichotoma*	Diterpene	-	[54]
**95**	Dictymal	*D. dichotoma*	Diterpene	-	[55]
**96**	Dichotenone A	*D. dichotoma*	Diterpene	-	[56]
**97**	Dichotenone B	*D. dichotoma*	Diterpene	-	[56]
**98**	Loliolide	*D. dichotoma*	Diterpene	-	[56]
**99**	Dichotenol A	*D. dichotoma*	Diterpene	-	[57]
**100**	Dichotenol B	*D. dichotoma*	Diterpene	-	[57]
**101**	Dichotenol C	*D. dichotoma*	Diterpene	-	[57]
**102**	Dictytriene A	*D. dichotoma*	Diterpene	-	[58]
**103**	Dictytriene B	*D. dichotoma*	Diterpene	-	[58]
**104**	Dictyone	*D. dichotoma*	Diterpene	-	[58]
**105**	Dictytriol	*D. dichotoma*	Diterpene	-	[58]
**106**	Tricyclodictyofuran A	*D. dichotoma*	Diterpene	-	[59]
**107**	Tricyclodictyofuran B	*D. dichotoma*	Diterpene	-	[59]
**108**	Tricyclodictyofuran C	*D. dichotoma*	Diterpene	-	[59]
**109**	Dictyoxetane	*D. dichotoma*	Diterpene	-	[60]
**110**	Pachydictyol B	*D. dichotoma*	Diterpene	-	[61]

**111**	Pachydictyol C	*D. dichotoma, D. spiralis*	Diterpene	Anti-trypanosomal, Cytotoxic	[61, 126]
**112**	Dictyol E	*D. dichotoma, D. fasciola, D. guineensis, D. spiralis*	Diterpene	Anti-trypanosomal, Cytotoxic	[61, 92, 99, 126]
**113**	*cis*-Africanan-1α-ol	*D. dichotoma*	Diterpene	-	[61]
**114**	Fucosterol	*D. dichotoma*	Sterol	Antiproliferative, Antimicrobial	[61, 63, 65]
**115**	2,2,6,7-Tetramethyl-10-oxatricyclo[4.3.0.1(1,7)]-decan-5-one	*D. dichotoma*	Oxatricyclic derivative	-	[61]
**116**	*N*-(4-bromo-*n*-butyl)-piperidin-2-one	*D. dichotoma*	Halogenated derivative	-	[61]
**117**	*tert*-Hexadecanethiol	*D. dichotoma*	Thiol derivative	-	[61]
**118**	Dictyotadiol	*D. dichotoma, D. guineensis*	Diterpene	-	[62, 99]
**119**	Coprostanol	*D. dichotoma*	Sterol	-	[63]
**120**	Epicoprostanol	*D. dichotoma*	Sterol	-	[63]
**121**	Campesterol	*D. dichotoma*	Sterol	-	[63]
**122**	Stigmasterol	*D. dichotoma*	Sterol	-	[63]
**123**	β-Sitosterol	*D. dichotoma*	Sterol	-	[63]
**124**	Cholesterol	*D. dichotoma*	Sterol	-	[63]
**125**	Brassicasterol	*D. dichotoma*	Sterol	-	[63]
**126**	Cholestanol	*D. dichotoma*	Sterol	-	[63]
**127**	5β-Cholestan-3-one	*D. dichotoma*	Sterol	-	[63]
**128**	1-Octanol	*D. dichotoma*	Aldehyde	-	[65]
**129**	p-Cresol	*D. dichotoma*	Aromatic derivative	-	[65]
**130**	2,6-Nonadienal	*D. dichotoma*	Aldehyde	-	[65]
**131**	Trans-anethole	*D. dichotoma*	Aromatic derivative	-	[65]
**132**	α-Cubebene	*D. dichotoma*	Diterpene	-	[65, 66]
**133**	β-Bourbonene	*D. dichotoma*	Diterpene	-	[65, 66]
**134**	β-Cubebene	*D. dichotoma*	Sesquiterpene	-	[65, 66]
**135**	γ-Gurjurene	*D. dichotoma*	Sesquiterpene	-	[65]
**136**	Germacrene D	*D. dichotoma*	Sesquiterpene	-	[65, 66]
**137**	α-Muurolene	*D. dichotoma*	Sesquiterpene	-	[65, 66]
**138**	α-Amorphene	*D. dichotoma*	Sesquiterpene	-	[65, 66]
**139**	δ-Cadenene	*D. dichotoma*	Sesquiterpene	-	[65, 66]
**140**	*cis*-Calamenene	*D. dichotoma*	Sesquiterpene	-	[65, 66]
**141**	α-Calacorene	*D. dichotoma*	Sesquiterpene	-	[65, 66]
**142**	β-Sesquiphellandrene	*D. dichotoma*	Sesquiterpene	-	[66]

**143**	α-Cadinol	*D. dichotoma*	Sesquiterpene	-	[66]
**144**	Cembrene	*D. dichotoma*	Sesquiterpene	-	[66]
**145**	Cycloisosativene	*D. dichotoma*	Sesquiterpene	-	[66]
**146**	τ-Muurolol	*D. dichotoma*	Sesquiterpene	-	[66]
**147**	α-Ylangene	*D. dichotoma*	Sesquiterpene	-	[66]
**148**	δ-Selinene	*D. dichotoma*	Sesquiterpene	-	[66]
**149**	α-Copaene	*D. dichotoma*	Sesquiterpene	-	[66]
**150**	Aromadendrene	*D. dichotoma*	Sesquiterpene	-	[66]
**151**	(E)-β-Farnesene	*D. dichotoma*	Hydrocarbon	-	[66]
**152**	α-Curcumene	*D. dichotoma*	Aromatic derivatives	-	[66]
**153**	Bicyclogermacrene	*D. dichotoma*	Sesquiterpene	-	[66]
**154**	Epizonarene	*D. dichotoma*	Sesquiterpene	-	[66]
**155**	γ-Cadinene	*D. dichotoma*	Sesquiterpene	-	[66]
**156**	γ-Muurolene	*D. dichotoma*	Sesquiterpene	-	[66]
**157**	Germacrene B	*D. dichotoma*	Sesquiterpene	-	[66]
**158**	*trans*-Cadina-1,4-diene	*D. dichotoma*	Sesquiterpene	-	[66]
**159**	1,8-Cineole	*D. dichotoma*	Sesquiterpene	-	[66]
**160**	6-Hydroxydolabella-3,7,12-triene	*D. dichotoma* var. *divaricata*	Diterpene	-	[67]
**161**	9-Hydroxyisodolasta-1,3,5(14)-trienone	*D. dichotoma* var. *divaricata*	Diterpene	Antiproliferative	[67, 72]
**162**	9-Hydroxydolasta-1,3-diene	*D. dichotoma* var. *divaricata*	Diterpene	-	[67]
**163**	3,4-Epoxy-6-hydroxydolabella-7,12-diene	*D. dichotoma* var. *divaricata*	Diterpene	-	[67]
**164**	12-Hydroxydolabella-3Z,7Z-dien-2-one	*D. dichotoma* var. *divaricata*	Diterpene	-	[67]
**165**	9,13-Dihydroxydolasta-1,3-diene	*D. dichotoma* var. *divaricata*	Diterpene	-	[67]
**166**	13-Acetoxy-9-hydroxydolasta-1,3-diene	*D. dichotoma* var. *divaricata*	Diterpene	-	[67]
**167**	9-Hydroxydolasta-1,3-dien-13-one	*D. dichotoma* var. *divaricata*	Diterpene	-	[67]
**168**	17,18:18,19-Bisepoxyxenic-methoxy-triene	*D. dichotoma* var. *divaricata*	Diterpene	-	[68]
**169**	3β-Hydroxydilophol	*D. dichotoma* var. *divaricata*	Diterpene	-	[68]
**170**	18-Hydroxy-2,7-dolabelladiene	*D. dichotoma* var. *divaricata*	Diterpene	-	[68]
**171**	4,17-Hydroxyxenic-trienaloic acid lactone	*D. dichotoma* var. *divaricata*	Diterpene	-	[69]
**172**	17-Xenic-trien-1-al-18-oic acid lactone	*D. dichotoma* var. *divaricata*	Diterpene	-	[69]

**173**	Epoxyxenic-hydroxydienaloic acid lactone	*D. dichotoma* var. *divaricata*	Diterpene	-	[69]
**174**	17-Acetoxyxenic-4-hydroxy-trien-dial	*D. dichotoma* var. *divaricata*	Diterpene	-	[69]
**175**	17-Acetoxy-4α-hydrocrenulide	*D. dichotoma* var. *divaricata*	Diterpene	-	[69]
**176**	Deacetoxydictyol H	*D. dichotoma* var. *divaricata*	Diterpene	-	[69]
**177**	2-Hydroxydictyoxide	*D. dichotoma* var. *divaricata*	Diterpene	-	[69]
**178**	(3α*S*,4α*R*,8*S*,8α*S*)-4α-Hydroxy-3α,8α-dimethyl-5-methylidene-1-(propan-2-yl)-3,3α,4,4α,5,6,7,8,8α,9-decahydrobenzo[f]azulen-8-yl acetate	*D. dichotoma* var. *divaricata*	Diterpene	-	[70]
**179**	(3α*S*,4α*R*,8*S*,8α*S*,10*S*)-4α,8-Dihydroxy-3α,8α-dimethyl-5-methylidene-1-(propan-2-yl)-2,3,3α,4,4α,5,6,7,8,8α,9,10-dodecahydrobenzo[f]azulen-10-yl acetate	*D. dichotoma* var. *divaricata*	Diterpene	-	[70]
**180**	(3α*S*,4α*R*,8*S*,8α*S*,10*S*)-4α-Hydroxy-3α,8α-dimethyl-5-methylidene-1-(propan-2-yl)-2,3,3α,4,4α,5,6,7,8,8α,9,10-dodecahydrobenzo[f]azulene-8,10-diyl diacetate	*D. dichotoma* var. *divaricata*	Diterpene	-	[70]
**181**	4α-Hydroxy-3α,8α-dimethyl-5-methylidene-1-(propan-2-yl)-2,3,3α,4,4α,5,6,7,8,8α,9,10-dodecahydrobenzo[f]azulen-10-yl acetate	*D. dichotoma* var. *divaricata*	Diterpene	-	[70]
**182**	Divarinone	*D. dichotoma* var. *divaricata*	Diterpene	-	[71]
**183**	Dolabellatrienol	*D. dichotoma* var. *implexa*	Diterpene	-	[72]
**184**	Amijiol acetate	*D. dichotoma* var. *implexa*	Diterpene	-	[72]
**185**	Dolastane amijiol-7-10-diacetate	*D. dichotoma* var. *implexa*	Diterpene	-	[72]
**186**	8β-Hydroxy-pachydictyol A	*D. dichotoma* var. *implexa*	Diterpene	-	[72]
**187**	Amijiol	*D. dichotoma* var. *implexa*	Diterpene	-	[72, 82]
**188**	Dictyol I acetate	*D. dichotoma* var. *implexa*	Diterpene	-	[73]

**189**	Indicol	*D. dichotoma* var. *indica*	Diterpene	Antiproliferative	[74]
**190**	Indicarol acetate	*D. dichotoma* var. *indica.*	Diterpene	-	[74]
**191**	Isolinearol	*D. dichotoma* var. *indica*	Diterpene	-	[74]
**192**	Linearol	*D. dichotoma* var. *indica*	Diterpene	-	[74]
**193**	Dictinol	*D. dichotoma* var. *indica*	Diterpene	-	[76]
**194**	Dictindiol	*D. dichotoma* var. *indica*	Diterpene	-	[76]
**195**	Dictintriol	*D. dichotoma* var. *indica*	Diterpene	-	[76]
**196**	Dictyotriol A	*D. dichotoma* var. *indica*	Diterpene	-	[77]
**197**	Dictyotriol B	*D. dichotoma* var. *indica*	Diterpene	-	[77]
**198**	Fucoxanthin	*D. dichotoma* var. *indica*	Carotenoid	Antiproliferative	[78, 79]
**199**	Isoamijiol	*D. dichotoma* var. *linearis.*	Diterpene	-	[82]
**200**	14-Deoxyamijiol	*D. dichotoma* var. *linearis*	Diterpene	-	[82]
**201**	Amijitrienol	*D. dichotoma* var. *linearis*	Diterpene	-	[83]
**202**	14-Deoxyisoamijiol	*D. dichotoma* var. *linearis*	Diterpene	-	[83]
**203**	4-Acetoxy-9,14-dihydroxydolastadiene	*D. dichotoma* var. *linearis*	Diterpene	-	[84]
**204**	14-Hydroxydolasta-1(15),7,9-triene	*D. dichotoma* var. *linearis*	Diterpene	-	[84]
**205**	4,9,14-Trihydroxydolasta-1(15),7-diene	*D. dichotoma* var. *linearis*	Diterpene	-	[84]
**206**	4,7,14-Trihydroxydolasta-1(15),8-diene	*D. dichotoma* var. *linearis*	Diterpene	-	[84]
**207**	4,6-Diacetoxy-14-hydroxydolastadiene	*D. dichotoma* var. *linearis*	Diterpene	-	[84]
**208**	Isopachydictyolal	*D. dichotoma* var. *linearis*	Diterpene	Antiviral	[85]
**209**	4α-Acetyldictyodial	*D. dichotoma* var. *lineari.*	Diterpene	Antiviral	[85]
**210**	2-Methyl-6-[(2S,3α*S*,4*R*,5*S*,7*R*,8α*R*)-2,4,7-trihydroxy-3,8-dimethylidenedecahydroazulen-5-yl]hept-2-ene-4,5-diyl diacetate	*D. dichotoma* var. *volubilis*	Diterpene	-	[86]

**211**	6-Methyl-2-[(2*S*,3α*S*,4*R*,5*S*,7*R*,8α*R*)-2,4,7-trihydroxy-3,8-dimethylidenedecahydroazulen-5-yl]hept-5-en-3-yl acetate	*D. dichotoma* var. *volubilis*	Diterpene	-	[86]
**212**	2-Methyl-6-[(3α*R*,4*R*,5*S*,7*R*,8α*R*)-3,4,7-trihydroxy-3-methyl-8-methylidene-3,3α,4,5,6,7,8,8α-octahydroazulen-5-yl]hept-2-ene-4,5-diyl diacetate	*D. dichotoma* var. *volubilis*	Diterpene	-	[86]
**213**	6-[(3α*R*,4*R*,5*S*,8α*R*)-3,4-Dihydroxy-3-methyl-8-methylidene-3,3α,4,5,6,7,8,8α-octahydroazulen-5-yl]-2-methylhept-2-en-4-yl acetate	*D. dichotoma* var. *volubilis*	Diterpene	-	[86]
**214**	2-[(3α*S*,4*R*,5*S*,7*R*,8α*R*)-4,7-Dihydroxy-3-methyl-8-methylidene-1,3α,4,5,6,7,8,8α-octahydroazulen-5-yl]-5-hydroxy-6-methylhept-6-en-3-yl acetate	*D. dichotoma* var. *volubilis*	Diterpene	-	[86]
**215**	6-[(3α*S*,4R,5*S*,7*R*,8α*R*)-4-(Acetyloxy)-7-hydroxy-3-methyl-8-methylidene-1,3α,4,5,6,7,8,8α-octahydroazulen-5-yl]-2-methylhept-2-ene-4,5-diyl diacetate	*D. dichotoma* var. *volubilis*	Diterpene	-	[87]
**216**	6-[(3α*S*,4*R*,5*S*,7*R*,8α*R*)-4,7-Dihydroxy-3-methyl-8-methylidene-1,3α,4,5,6,7,8,8a-octahydroazulen-5-yl]-2-methylhept-2-ene-4,5-diyl diacetate	*D. dichotoma* var. *volubilis*	Diterpene	-	[87]
**217**	(4E)-2-[(3α*S*,4*R*,5*S*,7*R*,8α*R*)-4,7-Dihydroxy-3-methyl-8-methylidene-1,3α,4,5,6,7,8,8α-octahydroazulen-5-yl]-6-hydroxy-6-methylhept-4-en-3-yl acetate	*D. dichotoma* var. *volubilis*	Diterpene	-	[87]
**218**	6-Methyl-2-[(3α*R*,4*R*,5*S*,7*R*,8α*R*)-3,4,7-trihydroxy-3-methyl-8-methylidene-3,3α,4,5,6,7,8,8α-octahydroazulen-5-yl]hept-5-en-3-yl acetate	*D. dichotoma* var. *volubilis*	Diterpene	-	[87]
**219**	Dilophol	*D. dichotoma* var. *volubilis*	Diterpene	-	[87]

**220**	Neodictyolactone	*D. fasciola*	Diterpene	-	[92]
**221**	18-Hydroxy-2,7-dolabelladiene	*D. fasciola*	Diterpene	-	[92]
**222**	Secospatane	*D. fenestrata*	Diterpene	-	[93]
**223**	2-Acetoxyspata-13(15),17-dien-10-ol	*D. fenestrata*	Diterpene	-	[93]
**224**	Dolabelladienetriol	*D. friabilis*	Diterpene	Antiviral	[94]
**225**	Pachydictyol-A epoxide	*D. furcellata*	Diterpene	-	[95]
**226**	Dictyodiol	*D. furcellata*	Diterpene	-	[96]
**227**	6,7-Diacetoxydolasta-1(15),8-dien-14-ol	*D. furcellata*	Diterpene	-	[98]
**228**	Hauckiosterol	*D. hauckiana*	Sterol	-	[100]
**229**	Hydroxydilophol	*D. masonii*	Diterpene	-	[102]
**230**	Dictyol K	*D. menstrualis*	Diterpene	Anti-inflammatory, Antimicrobial	[103]
**231**	Dictyol M	*D. menstrualis*	Diterpene	Anti-inflammatory,	[103]
**232**	Dictyol N	*D. menstrualis*	Diterpene	Antimicrobial	[103]
**233**	Isoacetoxycrenulatin	*D. menstrualis*	Diterpene	Anti-inflammatory,	[103]
**234**	4-Hidroxycrenulide	*D. menstrualis*	Diterpene	Antimicrobial	[103]
**235**	(6*R*)-6-Hydroxydichotoma-3,14-diene-1,17-dial	*D. menstrualis*	Diterpene	Antiviral	[104]
**236**	(6*R*)-6-Acetoxidichotoma-3,14-diene-1,17-dial	*D. menstrualis*	Diterpene	Antiviral	[104]
**237**	Dichotomanol	*D. menstrualis*	Diterpene	Anticoagulant, Antiviral	[105]
**238**	8,10,18-Trihydroxy-2,6-dolabelladiene	*D. menstrualis*	Diterpene	Antiviral	[106]
**239**	Dictyol L	*D. pinnatifida*	Diterpene	Antimicrobial	[113]
**240**	6-Epipachydictyol A	*D. pinnatifida*	Diterpene	Anti-microbial	[113]
**241**	6-Epidictyol C	*D. pinnatifida*	Diterpene	Anti-microbial	[113]
**242**	18-Acetoxy-xenianol	*D. pinnatifida*	Diterpene	Anti-microbial	[113]
**243**	4α-Hydroxyisodictyohemiacetal	*D. plectens*	Diterpene	Antiviral	[114]
**244**	4α-Hydroxyisodictyoacetal	*D. plectens*	Diterpene	Antiviral	[114]
**245**	13,18-Diacetoxy-4-hydroxyisodictyo-19-al	*D. plectens*	Diterpene	Antiviral	[114]
**246**	4α-Hydroxypachylactone	*D. plectens*	Diterpene	Antiviral	[114]
**247**	Isodictyohemiacetal	*D. plectens*	Diterpene	Antiviral	[114]
**248**	Isodictyoacetal	*D. plectens*	Diterpene	Antiviral	[114]
**249**	(2*S*,3*S*,4*R*,10*R*,19*R*)-19-Deoxo-4-hydroxy-19-methoxydictyolactone	*D. plectens*	Diterpene	Antiviral	[114]
**250**	4-Hhydroxydictyolactone	*D. plectens*	Diterpene	Antiviral	[114]

**251**	(1*S*,2*S*,3*E*,5*Z*,7*E*,11*R*,12*R*)-2-Acetoxy-12-hydroxydolabella-3,5,7-trien-9-one	*D. plectens*	Diterpene	-	[115]
**252**	(1*S*,2*S*,3*E*,5*Z*,7*Z*,11*R*,12*R*)-2-Acetoxy-12-hydroxydolabella-3,5,7-trien-9-one	*D. plectens*	Diterpene	-	[115]
**253**	(1*S*,2*S*,3*S*,4Z,6Z,8*R*,11*R*,12*R*)-2-Acetoxy-12-hydroxydolasta-4,6-dien-9-one	*D. plectens*	Diterpene	-	[115]
**254**	9α-Hydroxydictyol E	*D. plectens*	Diterpene	-	[115]
**255**	Isodictyol E	*D. plectens*	Diterpene	-	[115]
**256**	3β-Acetoxydilophol	*D. plectens*	Diterpene	-	[115]
**257**	19-Acetyl-4-hydroxydictyodiol	*D. plectens*	Diterpene	-	[115]
**258**	Hydroxydictyodial	*D. spinulosa*	Diterpene	Antifeedant	[118]
**259**	Spiralyde A	*D. spiralis*	Diterpene	Anti-leishmanial, Anti-trypanosomal	[119]
**260**	(1*R*,3*S*,4*S*,7*E*,11*S*,12*S*)-3,4-Epoxy-7,18-dolabelladiene	*D. spiralis*	Diterpene	Anti-leishmanial, Anti-trypanosomal	[119]
**261**	(1*R*,3*S*,4*S*,7*E*,11*S*,12*S*,14*S*)-3,4-Epoxy-14-hydroxy-7,18-dolabelladiene	*D. spiralis*	Diterpene	Anti-leishmanial, Anti-trypanosomal	[119]
**262**	(1*R*,3*S*,4*S*,7*E*,11*S*,12*S*)-3,4-Epoxy-14-oxo-7,18-dolabelladiene	*D. spiralis*	Diterpene	Anti-leishmanial, Anti-trypanosomal	[119]
**263**	(1*R*,3*E*,7*E*,11*S*,12*S*)-14-Oxo-3,7,18-dolabellatriene	*D. spiralis*	Diterpene	Anti-leishmanial, Anti-trypanosomal	[119]
**264**	(1*R*,3*Z*,7*E*,11*S*,12*S*)-14-Oxo-3,7,18-dolabellatriene	*D. spiralis*	Diterpene	Anti-leishmanial, Anti-trypanosomal	[119]
**265**	Dolabellane	*Dictyota sp.*	Diterpene	-	[120]
**266**	Xenicane	*Dictyota sp.*	Diterpene	-	[120]
**267**	Prenylated guaiane	*Dictyota sp.*	Diterpene	-	[120]
**268**	3,4-Epoxy-14-oxo-7,18-dolabelladiene	*Dictyota sp.*	Diterpene	Antimicrobial	[120]
**269**	Acetoxycrenulide	*Dictyota sp.*	Diterpene	Antimicrobial	[120]
**270**	10,18-Dihydroxydolabella-2,7-diene	*Dictyota sp.*	Diterpene	-	[120]
**271**	10-Acetoxy-18-hydroxydolabella-2,7-diene	*Dictyota sp.*	Diterpene	Antimicrobial	[120]
**272**	1-*O*-Octadecenoylglycerol	*Dictyota sp.*	Diterpene	Antimicrobial	[120]
**273**	sn-3-*O*-(Geranylgeranyl) glycerol	*Dictyota sp.*	Diterpene	Antimicrobial	[120]
**274**	Dictyotadimer A	*Dictyota sp.*	Diterpene	-	[121]
**275**	Joalin	*Dictyota sp.*	Diterpene	-	[122]
**276**	16-Acetoxy-1*R*,11*S*,12*R*-dolabella-triene	*Dictyota sp.*	Diterpene	-	[123]

**277**	3*S*-Acetoxy-1*R*,11*S*,12*R*-dolabella-triene	*Dictyota sp.*	Diterpene	-	[123]
**278**	Dictyotriol C	*Dictyota sp.*	Diterpene	-	[124]
**279**	Dictyotriol D	*Dictyota sp.*	Diterpene	-	[124]
**280**	Dictyotriol E	*Dictyota sp.*	Diterpene	-	[124]
**281**	α-Dictalediol monoacetate	*Dictyota sp.*	Diterpene	-	[125]

Table S2: Biological assays related to therapeutic potential of *Dictyota* extracts

No.	Name	Species	Activity	References
**1**	Chloroform and acetone extract	*D. acutiloba*	Antimicrobial	[12]
**2**	Methanol and hexane extract	*D. acutiloba*	-	[12]
**3**	Aqueous extract	*D. bartayresiana*	Antifungal	[14]
**4**	Methanol extract	*D. bartayresiana*	Antimicrobial	[15]
**5**	Methanol extract	*D. bartayresiana*	Larvicidal	[15]
**6**	Methanol extract	*D. bartayresiana*	Antioxidant	[16]
**7**	SiO_2–ZnO nanoparticles	*D. bartayresiana*	Antimicrobial, Antiproliferative	[17]
**8**	Sulfated polysaccharides	*D. caribaea*	Antiproliferative, Immunostimulatory	[19, 20]
**9**	Ethanol extract	*D. coriacea*	Hypopigmentation	[27]
**10**	Hexane and ethyl acetate fractions	*D. coriacea*	Anti-alopecia	[28]
**11**	Ethanol extract	*D. coriacea*	Anti-inflammatory	[29]
**12**	Polysaccharides	*D. dichotoma*	Antiproliferative, Immunomodulator, Antiviral	[36-38]
**13**	Ethanol extract	*D. dichotoma*	Larvicidal, Antioxidant	[39, 42]
**14**	Chloroform fraction	*D. dichotoma*	Antioxidant Antiproliferative	[42]
**15**	Dichloromethane extract	*D. dichotoma*	Antioxidant	[65]
**16**	Palladium oxide nanoparticles	*D. dichotoma* var. *indica*	-	[79]
**17**	Gold nanoparticles	*D. dichotoma*	Antimicrobial	[81]
**18**	Methanol extract	*D. dichotoma* var. *divaricata* *D. dichotoma* var. *linearis*	Cytotoxic	[84]
**19**	Aqueous and ethanolic extracts	*D. dichotoma*	Antioxidant, Plant growth promotion	[88, 89]
**20**	Methanol extract	*D. dichotoma* var. *intricata,* *D. dichotoma* var. *indica,* *D. dumosa*	Antimicrobial	[90]
**21**	Dichloromethane and dichloromethane/methanol extracts	*D. fasciola*	Antimicrobial	[92]
**22**	*n*-Butanol extract	*D. flabellate*	Antimicrobial, Antioxidant	[97]

**23**	Ethanol extract	*D. dichotoma* var. *indica,* *D. hauckiana*	Larvicidal	[101]
**24**	Dichloromethane/methanol-soluble fraction	*D. menstrualis*	Antiviral	[104]
**25**	Methanol extract	*D. cilliolata,* *D. menstrualis*	Antiproliferative	[107]
**26**	Sulfated polysaccharides	*D. menstrualis*	Anticoagulant, Antinociceptive, Anti-inflammatory	[108, 109]
**27**	Dichloromethane extract	*D. menstrualis*	Antiviral	[110]
**28**	Sulfated polysaccharides	*D. mertensii*	Antiproliferative, Immunomodulatory, Anti-inflammatory	[111]
**29**	Dichloromethane methanol extract	*D. pulchella*	Cardioprotective	[116]
**30**	Dichloromethane extract	*D. spiralis*	Anti-leishmanial, Anti-trypanosomal	[119]
**31**	Organic extract	*D. spiralis*	Anti-leishmanial, Anti-trypanosomal	[126]
**32**	Organic extract	*Dictyota sp.*	Antioxidant, Neuroprotective	[127]
